# Cyprinid herpesvirus 2 infection changes microbiota and metabolites in the gibel carp (*Carassius auratus gibelio*) midgut 

**DOI:** 10.3389/fcimb.2022.1017165

**Published:** 2023-02-02

**Authors:** Peng Chen, Mingming Zhang, Yichan Zhang, Jun Li, Xihe Wan, Tingli Lv, Yiyue Chen, Zhigang Zhao, Zhihao Ma, Zhu Zhu, Lihua Chen, Zhen Li, Zisheng Wang, Guo Qiao

**Affiliations:** ^1^ Research Center of Aquatic Animal Immunity and Disease Control, Yancheng Institute of Technology, Yancheng, Jiangsu, China; ^2^ Heilongjiang Provincial Key Laboratory of Cold Water Fish Germplasm Resources and Aquaculture, Heilongjiang River Fisheries Research Institute, Chinese Academy of Fishery Sciences, Harbin, China; ^3^ Department of Biological Sciences, Biomolecular Sciences Institute, Florida International University, Miami, FL, United States; ^4^ Central Key Laboratory of Jiangsu Institute of Marine Fisheries, Nantong, Jiangsu, China; ^5^ Center of Fisheries technology popularization Sheyang Agricultural and Rural Bureau, Yancheng, Jiangsu, China; ^6^ College of Agricultural Science and Engineering, Hohai University, Nanjing, China

**Keywords:** CyHV-2, gut microbiota-mediated metabolites, *Aeromonas*, *Cetobacterium*, tryptophan metabolism

## Abstract

Cyprinid herpesvirus 2 (CyHV-2) infects gibel carp (*Carassius auratus gibelio*) and causes severe losses. Microbiota in animal guts involves nutrition intake, development, immunity, and disease resistance. However, the relationship between gibel carp gut microbiota and CyHV-2 infection is not well known. Herein, we analyzed the gut microbiota composition and metabolite profiles in CyHV-2-infected and -uninfected fish using high-throughput sequencing and gas chromatography/mass spectrometry. Results showed that CyHV-2 infection significantly changed gut microbiota and metabolite profiles (*p* < 0.05). High-throughput sequencing demonstrated that the relative abundance of *Aeromonas* in the midgut increased dramatically while *Cetobacterium* decreased. Time-course analysis showed that the number of *Aeromonas* in the midgut of infected fish increased more than 1,000 times within 5 days post infection. Metabolome analysis illustrated that CyHV-2 infection significantly altered 24 metabolites in the midgut of gibel carp, annotating to the anomaly of digestion and metabolisms of amino acids, carbohydrates, and lipids, such as tryptophan (Trp) metabolism. The Mantel test demonstrated that gut microbiota and metabolite profiles were well related (r = 0.89). Furthermore, Trp metabolism responded to CyHV-2 infection closely was taken as one example to prove the correlation among CyHV-2 infection, metabolites and microbiota in the midgut, and host immunity. Results showed that modulating Trp metabolism could affect the relative abundance of *Aeromonas* in the midgut of fish, transcription of antiviral cytokines, and CyHV-2 infection. Therefore, we can conclude that CyHV-2 infection significantly perturbed the gut microbiome, disrupted its’ metabolic functions, and caused the proliferation of the opportunistic pathogen *Aeromonas*. This study also suggests that modulation of the gut microbiome will open a therapeutic opportunity to control CyHV-2 infection in gibel carp.

## 1 Introduction

Gibel carp (*Carassius auratus gibelio*), an omnivorous freshwater fish, is one of the leading aquaculture species worldwide. More than 2,700,000 tons of gibel carps were produced in 2020 with an economic value of over 5 billion dollars (Ministry of Agriculture and Rural Affairs of China, 2021), and most of its production farms are located in Yancheng, Province Jiangsu, China (Liu et al., 2018). However, the disease caused by Cyprinid herpesvirus 2 (CyHV-2), also known as the goldfish hematopoietic necrosis virus (GFHNV), limits the sustainable aquaculture of gibel carp (Lu et al., 2022; Zhu et al., 2022). CyHV-2 infection in gibel carp was first reported in 1999 in China (Chang et al., 1999), and the massive mortality reached over 90% in many breeding ponds (Wang et al., 2012). The CyHV-2-infected fish displays lethargic, anorexic, and widespread bleeding in the body, gill, and swim bladder. When the gill cover opens and closes or the fish’s body is stressed or jumping, blood will flow out from the gills (some infected fish may not have gill-bleeding symptoms). Some infected fish have erythema on the gill cover, which is called a beauty spot. Internal organs are congested or bleeding, the fish’s swim bladder has obvious bleeding points compared to the hemorrhages caused by *Aeromonas* spp., and the tail fin is whitish (Wu et al., 2013). CyHV-2 can be naturally detected in the water and sediments in the culture ponds, and gibel carp is sensitive to this virus (Yao et al., 2016). Gills are the critical organ for the initial invasion of CyHV-2 into fish, while the kidney and spleen are the primary organs for viral replication (Ding et al., 2014; Zhu et al., 2015; Yao et al., 2016). However, there is still no effective strategy to control this infection, which threatens this species’ sustainable culture industry.

Recent studies reveal that modulating the gut microbiota is an effective strategy to control disease development in human beings (Liu et al., 2013), but few studies were reported on the fish (Ran et al., 2021). Gut microbiota is considered as a good indicator of animal health, and plays a vital role in regulating host immune homeostasis and metabolic function (Guarner and Malagelada, 2003; Hu, 2017). The following four roles related to gut microflora have been reported: 1) metabolic activities in the fermentation of non-digestible dietary residue and endogenous mucus: the salvage of energy as short-chain fatty acids, the production of vitamin K, and the absorption of ions; 2) critical trophic effects on intestinal epithelial cell growth and differentiation, and interactions between intestinal bacteria and host immunity at the mucosal interface; 3) protection or mucous integrity-dependent barrier against pathogen invasion by alien microbes; and 4) relation to certain pathological disorders, including multisystem organ failure and inflammatory diseases (Guarner and Malagelada, 2003; Shapira, 2016; Cong et al., 2022). Thus, determining causal links of gut microbiota to diseases can contribute to developing new therapeutic interventions (Guarner and Malagelada, 2003; Li et al., 2017; Agus et al., 2018).

Although the gut microbiota of fish is affected by many factors, including host factors (e.g., genetics, gender, weight, and immunity), environmental factors (e.g., water, salinity, diet, and medicine), and microbial factors (Wang et al., 2018), the composition of the gut core microbiota is relatively stable in fish (Wang et al., 2018). Among them, water, diet, and feeding habits have been studied the most. *Aeromonas*, *Pseudomonas*, and *Bacteroides* type A dominate the gut microbiota of freshwater fish species. *Plesiomonas*, *Enterobacteriaceae*, *Micrococcus*, *Acinetobacter*, *Clostridium*, *Bacteroides* type B, and *Fusariumas* are less abundant. Additionally, many studies have used the combined metabolomic and metagenomic methods to investigate the correlation between metabolic alterations and gut microbiota changes, providing new insights into the disease pathology and therapeutic intervention (Guarner and Malagelada, 2003; Wang et al., 2018). For gibel carp, the gut microbiota of fish post-CyHV-2 infection was changed, and *Plesiomonas* was highly abundant in the infected fish Rong et al., 2017. CyHV-2 and CyHV-3 infections ruin the gut integrity of carps. These findings raised the possibility of the direct interaction between CyHV-2 and commensal bacteria (Rong et al., 2017; Ran et al., 2021). However, few studies have been conducted to investigate the effect of CyHV-2 infection on the gut microbial ecology by using combined metabolomic and metagenomic analysis. It will hinder the control and prevention development of CyHV-2 infection in gibel carp culture.

Thus, in this study, the changes of microbiota and metabolites in the midgut of CyHV-2-infected gibel carp and the correlation among them were firstly investigated. Then, the time-dependent number changes of target bacteria associated with CyHV-2 infection were analyzed through the culture method. Furthermore, tryptophan (Trp) metabolism was taken as one of the examples to prove the correlation between CyHV-2 infection and the metabolites since 1) the changes of microbiota and metabolites in the fish midgut post-CyHV-2 infection in this study had been annotated to Trp metabolism; 2) Trp metabolism has been reported to play a crucial role in gut microbiota–host cross-talk in health and disease *via* the aryl hydrocarbon receptor (AhR) (Hao and Whitelaw, 2013; Agus et al., 2018; Natividad et al., 2018; Sun et al., 2019; Wyant and Moslehi, 2022); 3) AhR, a nuclear protein upon association with certain endogenous and exogenous ligands, can translocate signals into the nucleus, bind DNA, and regulate gene expression. Trp metabolites are one of the most important endogenous AhR ligands (Ghiboub et al., 2020; Willem et al., 2022); and 4) Trp metabolism is known to regulate the intestinal barrier function and host immunity, and Trp metabolites/AhR signaling modulation are a therapeutic perspective in human diseases (Lamas et al., 2016; Lamas et al., 2018; Trikha and Lee, 2019; Platten et al., 2019; Ghiboub et al., 2020; Rosser et al., 2020). Taken together, these results about microbiome-modulated metabolites from this study will provide valuable information to control CyHV-2 infection from a disrupted equilibrium to clinical opportunities.

## 2 Materials and methods

### 2.1 Ethics statement

In the present study, all the animal experiments were handled in accordance with the guidelines for the Care and Use of Experimental Animals of China. The Committee for the Welfare and Ethics of Laboratory Animals was approved by the Animal Care and Use Committee of the Center for Applied Aquatic Genomics at the Chinese Academy of Fishery Sciences.

### 2.2 Fish acclimation

Gibel carp (43.17 ± 2.9 g) was obtained from a fish farm at Yancheng City, Jiangsu province China. After bringing the fish to the lab, we first performed PCR to make sure that the fish did not carry the CyHV-2 and then did further research (**Figure S1**). The primers for PCR are listed in **Table S1**. PCR in the reaction volume of 20 μl was performed following the protocols as one cycle of 95°C for 5 min, followed by 30 cycles at 95°C for 30 s, 58°C for 30 s, 72°C for 30 s, and final elongation at 72°C for 5 min (Zhu et al., 2015). The PCR products were checked by 1% agarose gel electrophoresis.

Then, the fish were acclimated in a tank with static water at a water temperature of 23°C–25°C, pH of 7.8–8.2, and dissolved oxygen (DO) higher than 5 mg L^−1^ and provided with 24-h continuous aeration circulation for 2 weeks prior to the experiments. Gibel carp was fed three times each day with commercial diets (Tongwei, Yancheng, China) to satiation. The diets contained 32.25% crude protein, 5.90% crude lipid, 1.18% calcium, and 1.23% total phosphorus.

### 2.3 CyHV-2 solution preparation and viral quantification

The CyHV-2 solution was prepared as the previous description by Qiao et al. (2020). In brief, the kidney from naturally CyHV-2-infected gibel carp with typical signs of hemorrhage in the gill and swim bladder was sampled, homogenized by mixing with 10 times volume of phosphate-buffered saline (PBS, 0.01 M, pH7.2), centrifuged at 12,000 × g for 30 min and filtered by a 0.45/0.22-μm biofilter to remove bacteria.

DNA from the kidney was extracted using the viral DNA extraction kit (TaKaRa BIO Co., Ltd., Dalian, China), and the extracted DNA was assessed and adjusted to 50 ng μl^−1^ by Biophotometer Plus (Eppendorf). The primers (CyHV-2-RT-F and CyHV-2-RT-R) for quantitative real-time PCR (qPCR) are listed in **Table S1**. The qPCR reactions were performed using the SYBR^®^ Premix ExTaq™ kit (Takara Bio Co., Ltd., Dalian, China) in a total volume of 25 μl, containing 12.5 μl SYBR Premix Ex Taq II (TaKaRa, Japan), 2 μl template DNA, 1 μl each primer and 8.5 μl ddH_2_O. Distilled water replacing the extracted DNA was set as a negative control for each new run. The thermal cycling condition was as follows: one cycle of 95°C for 3 min, followed by 40 cycles at 95°C for 5 s and 55°C for 30 s and then by dissociation curve analysis (65°C–95°C: increment 0.5°C for 5 s) (Qiao et al., 2020). After amplification, melting curve analysis was performed, and Ct values were obtained. The standard curve of the viral DNA copy number is shown in **Figure S2**, which was generated as described by Xu et al. (2014). Plasmid DNA containing the sequence of the CyHV-2 helicase gene was selected to serve as the standard for virus quantification. The amplified DNA fragment was gel-purified using a Fermentas Gel Extraction Kit (Thermo). The resulting DNA fragment was inserted into the pMD19-T plasmid to produce pMD-CyHV-2. A 10-fold dilution series of pMD-CyHV-2 was used as the standard template of CyHV-2 in the qPCR. Finally, a viral stock solution at a concentration of 9.7 × 10^7^ copies μl^-1^ was prepared.

### 2.4 Fish infection with CyHV-2 through gills (simulated to natural infection)

The gut microbiota diversity of gibel carp is related to the environment, diets, and feeding habits (Chen et al., 2021), but the composition of core microbiota in the gut is relatively stable under the same cultural circumstance (Wang et al., 2018; Chen et al., 2021). Therefore, allogynogenetic gibel carp, after acclimation for 14 days in the lab, was house-infected by CyHV-2 to avoid the effect of gender, water, and diet on gut microbiota. After acclimation in part 2.2, fish were divided randomly into two groups, namely, a control group with PBS and the CyHV-2-infection group. Each group included six tanks, three of them (volume of 100 L) with 8 individuals were used to calculate cumulative mortality (CM) and another three (volume of 400 L) with 30 individuals was set to sample for gut microbiota and metabolite analysis.

The ‘per-gill infection method’ mocked natural infection, which directly exposes virus to only gills, had been used to infect gibel carp like KHV and CHNV (Miyazaki et al., 2008; Somamoto et al., 2015) since gills are one of the first organs of aquatic animals exposed to pathogens (Murray et al., 2017; Andrews et al., 2010) and key invasion portal for CyHV-2 in gibel carp (Ding et al., 2014). Briefly, fish were anesthetized with tricaine methane sulfonate (MS-222) at 100 mg L^−1^ and inoculated with a CyHV-2 working solution (9.7×10^6^ copies μl^−1^) at 10 μl fish^-1^ into their gills (both sides) in air. An equal volume of PBS was inoculated into gills as the control group. After gill exposure to the viral solution or PBS, fish were wrapped with wet papers and kept in air for 5–7 min at 25°C to allow the virus to adsorb into the gill tissue. Then, the fish were returned to the tank and maintained at 25°C with 24-h continuous aeration circulation. During the challenge test, no diets were fed (Qiao et al., 2020).

### 2.5 CyHV-2 infection confirmation

#### 2.5.1 Clinical symptom observation and cumulative mortality

Clinical symptoms were observed daily, focusing on lethargy, anorexia, and hemorrhage in the body, eye, gill, and swim bladder. CM within 14 days post infection (dpi) was counted.

#### 2.5.2 CyHV-2 load measurement

The fish with typical hemorrhagic symptoms at the 5th dpi were sampled. The viral load at 5 dpi in different tissues, including the gill, foregut, midgut, hindgut, liver, kidney, spleen, brain, and muscle, was detected by qPCR as described in part 2.3. Viral load was converted into copies per ng DNA.

### 2.6 Microbiome analysis of fish gut post-CyHV-2 infection

#### 2.6.1 Midgut microbiota analysis through high-throughput sequencing

The midgut of three individuals with typical hemorrhagic symptoms at 5 dpi from each tank was collected and pooled as one sample. Resultantly, a total of nine fish for each group and three biological replicates were conducted for gut microbiome analysis. Microbial DNA was extracted from the midgut using the HiPure Soil DNA kits (Magen, Guangzhou, China) according to the manufacturer’s protocols. DNA samples were amplified to target the V4 region of bacterial 16S rRNA (Qiao et al., 2020). PCR products were purified with Qiagen Gel Extraction Kit (Qiagen, Suzhou, China). The primers 341F and 806R were used, and sequences are listed in **Table S1** (Guo et al., 2017). Purified amplicons were pooled in the equimolar and paired-end sequences on an Illumina platform (MiSeq, Illumina Inc., Guangzhou, China) (2 × 250) following the standard protocols. The effective tags were clustered into operational taxonomic units (OTUs) of ≥97% similarity using the UPARSE (version 9.2.64) pipeline (Edgar, 2013). The tag sequence with the highest abundance was selected as a representative sequence within each cluster. A Venn diagram using the R package (version 1.6.16) to analyze and draw plots was performed in R project UpSetR package (version 1.3.3) to identify unique and common OTUs (Conway et al., 2017). The species comparison between groups was calculated by Welch’s *t*-test. Principal component analysis (PCA) was performed to distinguish the gut microbiota profiles between control and CyHV-2-infection groups. All α-diversity indexes were calculated in QIIME (version 1.9.1), and the alpha index comparison between groups was calculated by Welch’s *t*-test (Caporaso et al., 2010). Sequence alignment was performed using muscle (version 3.8.31), and phylogenetic tree was constructed using FastTree (version 2.1) (Edgar, 2004; Price et al., 2010); then, the weighted UniFrac distance matrix was generated by R project GuniFrac package (version 1.0) (Lozupone and Knight, 2006). The Kyoto Encyclopedia of Genes and Genomes KEGG pathway analysis of the OTUs was inferred using PICRUSt (version 2.1.4), and the analysis of functional difference between groups was calculated by Welch’s *t*-test (Langille et al., 2013). Canonical correspondence analysis (CCA) was executed in the R Project Vegan package (version 2.5.3) to clarify the influence of treatment on community composition (Oksanen et al., 2011). The biomarker features in each group were screened by the CCA effect size (LEfSe) software (version 1.0) (Edgar, 2013). Student’s *t*-test was used to evaluate the statistical differences of biological parameters between control and CyHV-2-infection groups. The significant difference was set at *p* < 0.05.

#### 2.6.2 Verification of time-dependent *Aeromonas* abundance within 5 dpi by the culture method

Based on the results from part 2.6.1 and *Aeromonas* frequent infection in gibel carp culture practice, the time-course abundance of *Aeromonas* in the midgut of fish after infected by CyHV-2 was detected. The midgut of fish at 0, 1, 4, and 5 dpi was collected and homogenized in precold PBS. Then, the homogenate was diluted and spread on the RS selective medium, which was cultured at 28°C for 24–48 h. Bacterial colonies were counted and converted into the CFU g^-1^ midgut. Meanwhile, the number of *Aeromonas* in culture water during the whole experiment was counted.

### 2.7 Metabolomic analysis of fish gut post-CyHV-2 infection

#### 2.7.1 Metabolomic analysis of fish midgut by Jiangsu Liquid Chromatography-Tandem Mass Spectrometry LC-MS/MS

The midgut from each fish with typical hemorrhagic symptoms at 5 dpi was sampled, cut off, and then flushed with PBS to collect metabolites. Six biological replicates were set for each group. LC-MS/MS analysis was performed using a Ultra High Performance Liquid Chromatography UHPLC system (1290, Agilent Technologies). Mass Spectrometer MS raw data (.raw) files were converted to the mzML format using ProteoWizard and processed by R package XCMS (version 3.2), including retention time alignment, peak detection, and peak matching. The OSI-SMMS (version 1.0, Dalian Chem Data Solution Information Technology Co. Ltd.) was used for peak annotation after data processing with an in-house MS/MS database. Partial least squares discriminant analysis (PLS-DA) is a supervised dimensionality reduction method and was applied in comparison groups using R package models (http://www.r-project.org/) (Worley and Powers, 2013). The metabolite resonances were identified according to the Human Metabolome Database (Deng et al., 2014). Significantly changed metabolites were identified based on the following criteria: fold change >2.00 or <0.56, VIP > 1, and *p* < 0.05.

#### 2.7.2 Detection of time-dependent concentration of aryl hydrocarbon receptor (AhR) within 5 days post-CyHV-2 infection

The results from parts 2.6 and 2.7.1 showed that 1) CyHV-2 infection increased the relative abundance of *Aeromonas* spp. and decreased *Cetobacterium* in the midgut significantly; 2) the concentration of indoleacetaldehyde decreased significantly; and 3) a strong positive correlation between them was also found. Additionally, indole derivatives such as indoleacetaldehyde are the products of Trp metabolism and activate the AhR signaling pathway and its downstream signals (Agus et al., 2018). AhR activation has been reported to play important roles in host health and diseases through modulating T-cell differentiation, the expression of cytokines, transcription factors, autoimmunity, and inflammation (Quintana et al., 2008; Kerkvliet et al., 2009; Apetoh et al., 2010; Jeremy et al., 2016). Thus, the time-dependent concentration of AhR in the midgut of infected fish at 0, 6, 24, 72, and 120 hpi was detected using the AhR assay kit (Jiangsu Jianglai Biotechnology Co. Ltd., Suzhou, China) according to the manufacturers’ protocol.

#### 2.7.3 Effect verification of *AhR* expression on CyHV-2 infection

6-Formylindolo[3,2-b]carbazole (FICZ), a Trp photoproduct postulated as a candidate physiological ligand of AhR (Rannug et al., 1987; Oberg et al., 2005; Jeong et al., 2012), was used to evaluate the effect of *AhR* expression on the bacterial diversity in the midgut, the expression of antiviral cytokines, and viral infection progress.

##### 2.7.3.1 Optimal FICZ working concentration assessment in gibel carp

FICZ (MedChemExpress, State of New Jersey USA) at the concentration of 1 μg fish^-1^ was injected by I.P., and an equal volume of PBS (100 μl fish^-1^) was injected as control. At 0, 12, 24, and 48 hpi, the fish midgut from two groups was sampled, immersed in RNA later (Sigma, Shanghai, China), and immediately stored at -80°C. Total RNA extraction, complementary DNA cDNA synthesis, and RT-qPCR were conducted as described in our previous study (Qiao et al., 2020). Briefly, RNA was extracted with the RNeasy mini kit (Qiagen, Valencia, CA, USA), and genomic DNA was erased through DNase I (Qiagen). A Nanodrop ND-1000 spectrophotometer was used to detect the quantity of RNA and concentration. Then, cDNA was synthesized using the oligo^dT^ primer and Prime-script™ first-strand cDNA synthesis kit (Takara Bio, Dalian, China). Genes *AhR1* and *AhR2* and downstream gene *Cyp1A1* (Cytochrome P450, family 1, member A1) were chosen to investigate the effect of FICZ on the expression of key genes involved in the AhR signaling pathway (Jeong et al., 2012). The gene-specific primers are listed in **Table S1**. The RT-qPCR was performed through SYBR^®^ Premix Ex Taq™ (Takara, Dalian, China) in a CFX96 Real-Time PCR Detection System (Bio-Rad, Shanghai, China) (Qiao et al., 2020). The relative fold changes of a specific gene in the FICZ injection group were compared to that in the control PBS-injected group using the 2^-ΔΔCt^ method (Livak and Schmittgen, 2001).

##### 2.7.3.2 Effect of FICZ on the expression of cytokines

FICZ was injected at 1 μg fish^-1^ by I.P., and an equal volume of PBS (100 μl fish^-1^) injected as control. At 12 hpi based on the results from part 2.7.3.1, the head kidney was sampled, immersed in RNA later, and immediately stored at -80°C. Total RNA extraction, cDNA synthesis, RT-qPCR, and the relative expression calculation of cytokines, including *IFN-γ* (interferon), *MX1* (myxovirus resistance 1), *ISG15* (interferon-stimulated gene 15), *JAK* (Janus kinase), *TNF-α* (tumor necrosis factor-α), *MAPK3* (mitogen-activated protein kinase 3), *IL-4* (interleukin-4), *Gata3* (Gata binding protein 3), and *T-Bet1* (T-box expressed in T cells 1), were performed as described in part 2.7.3.1. The genes and gene-specific primers are listed in **Table S1**.

##### 2.7.3.3 Effect of FICZ on the cumulative mortality of fish post-CyHV-2 infection

There were four groups, namely, the PBS single-injection group (10 μl fish^-1^), FICZ single-injection group, ‘FICZ+CyHV-2’-infection group, and ‘PBS+CyHV-2’- infection group. FICZ (1 μg fish^-1^) was injected into fish by I.P., PBS, and CyHV-2 immersed fish through the ‘per-gill’ method as described in part 2.4. Additionally, two viral concentrations were set, which were 9.7×10^6^ and 2.4×10^7^ copies μl^−1^. Each group included triplicates. Death within 14 days was recorded, and CM was calculated.

##### 2.7.3.4 Effect of FICZ on the viral replication *in vivo* post-CyHV-2 infection

The fish from both the FICZ (1 μg fish^-1^, I.P.) group and control group (PBS, 100 μl fish^-1^) were infected by CyHV-2 through the ‘per-gill’ method as described in part 2.4. At 6, 12, 24, 48, 72, and 120 hpi, the kidney from ‘FICZ +CyHV-2-infected’ fish and ‘PBS +CyHV-2-infected’ fish was sampled, and viral load was quantified by qPCR as described in part 2.3.

### 2.8 Correlation between the gut microbiome and metabolites

Pearson correlation coefficient between gut microbial composition and experimental treatment was calculated with the R-project psych package (version 1.8.4) (Hornik, 2012). A network of correlation coefficients was generated using Omicsmart, a dynamic real-time interactive online platform for data analysis (http://www.omicsmart.com). In order to further prove the change of metabolites on the relative abundance change of main bacteria species, the effect of *AhR* expression on the relative abundance of *Aeromonas* in the midgut of fish post-CyHV-2 infection was investigated. Briefly, based on the results from part 2.7.3.1, the optimal working model of FICZ *in vivo* was injected by I.P. at 1 μg fish^-1^ for 12 h. Four groups were set, including ‘PBS+ PBS’ single injection group (100 μl fish^-1^), ‘FICZ+ PBS’ injection group, ‘FICZ+CyHV-2’ injection group, and ‘PBS+CyHV-2’ injection group. FICZ and PBS were injected into fish by I.P. and CyHV-2-infected fish through the ‘per-gill’ method as described in part 2.4. They were conducted at 12-h intervals. At 0, 1, 4, 5, and 7 dpi, the midgut of fish in four groups was sampled and homogenized in precold PBS. Then, the homogenate was diluted and spread on TSA and RS-selective medium, which was cultured at 28°C for 24–48 h. The TSA medium was used to count the total culturable bacteria, and the RS medium was used for *Aeromonas* spp. Bacterial colonies were counted and converted into the CFU g^-1^ midgut.

### 2.9 Statistical analysis

Results are expressed as mean ± standard deviation and subjected to one-way analysis of variance using the statistical software program Statistical Product and Service Solutions SPSS version 17.0 (SPSS Inc., IL, USA). The significant difference was determined using Tukey’s multiple comparison test for more than three experimental groups and Student’s *t*-test for two experimental groups. Statistical significance was considered at P-values less than 0.05, and the results were expressed as mean ± SE (standard error).

## 3 Results

### 3.1 Viral load *in vivo* post-CyHV-2 infection through ‘per-gill’ method

**Figure 1:**
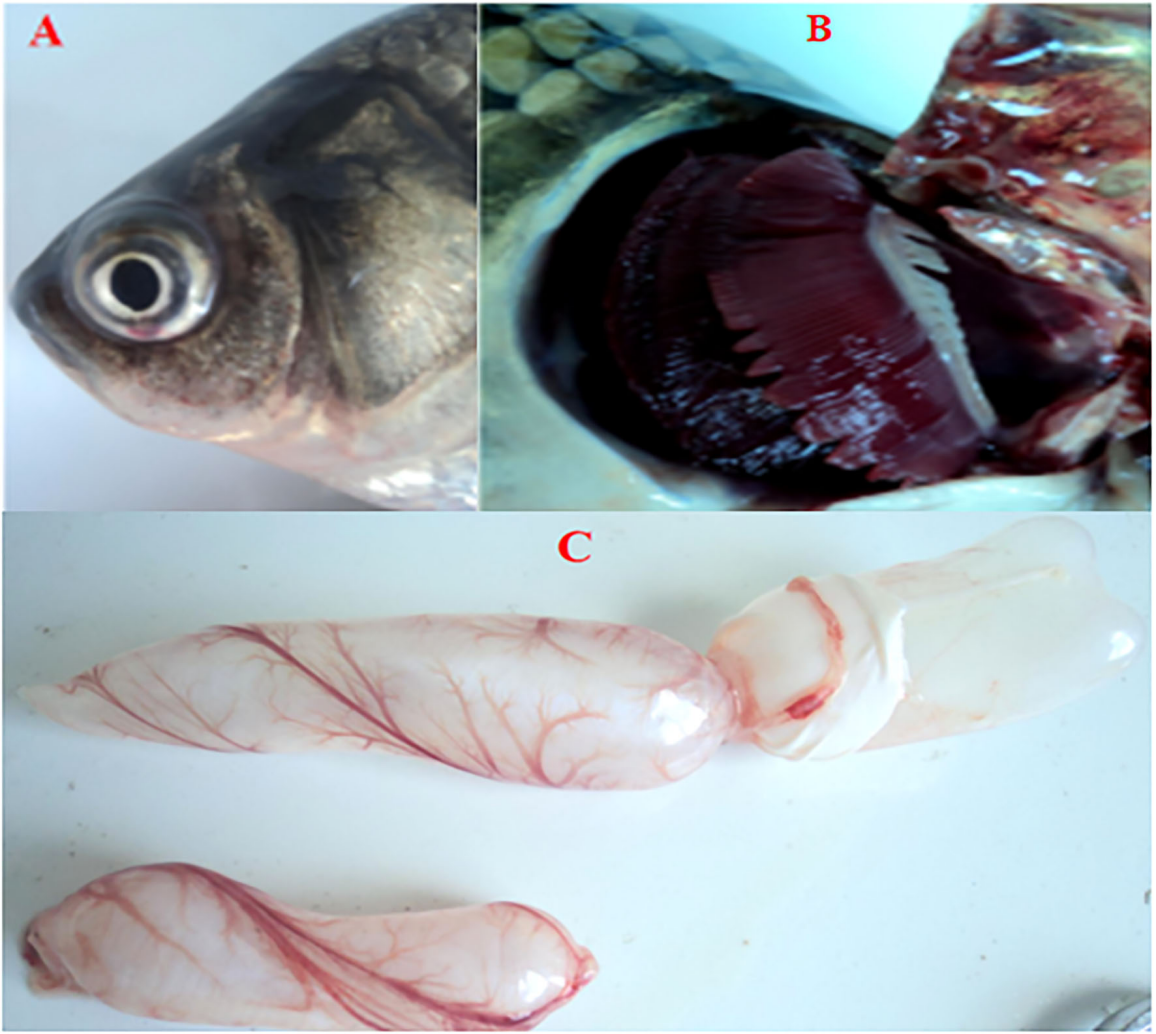
Cross-pathological signs of artificially infected gibel carp (*Carassius auratus gibelio*) by Cyprinid herpesvirus 2 (CyHV-2) through the per-gill method. **(A)** Severe hemorrhages on the body surface and bulging eye; **(B)** severe hemorrhages on gills; and **(C)** a petechial hemorrhage presented on the bladder wall.

**Figure 2:**
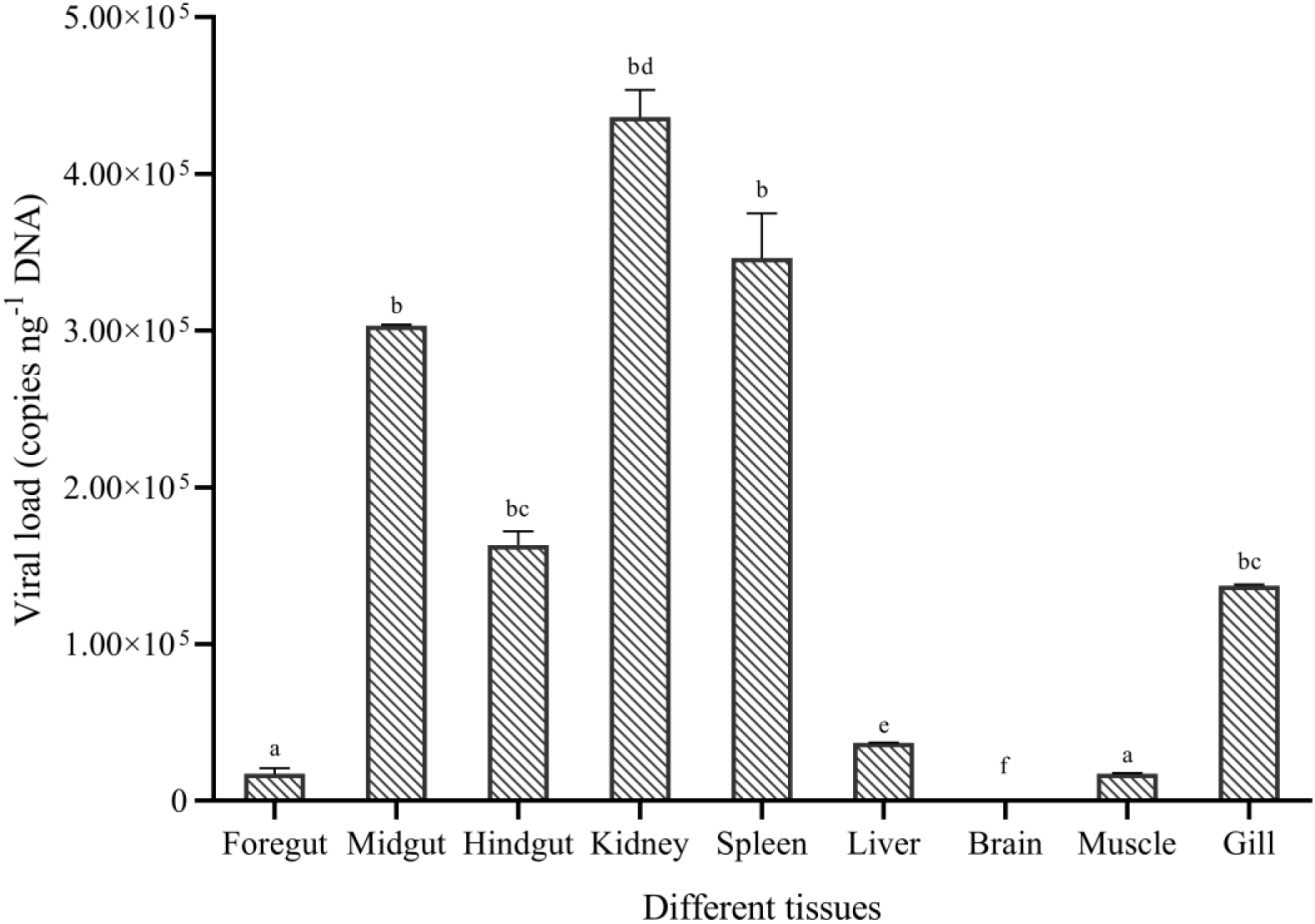
Viral replication in different tissues of gibel carp (*C. auratus gibelio*) challenged by CyHV-2 using the quantitative real-time PCR method. One-way ANOVA was conducted with SPSS 17.0 software. Different letters mean significant difference among tissues (*p* < 0.05).

No CyHV-2 was detected by PCR in the fish before the experiment (**Figure S1A**). Gibel carp could be infected by CyHV-2 through the ‘per-gill’ method in the lab. Like natural infection, hemorrhage in the fish skin and bulging eyes was observed at 5 dpi (**Figure 1A**). Severe hemorrhage was observed in the gills and swim bladder wall (**Figures 1B, C** **)**. In the control group, no CyHV-2 was detected and no clinical symptoms were observed (**Figures S1A, B**). CM in the CyHV-2-infection (9.7 × 10^7^ copies per fish) group was 50%, and no mortality was observed in the PBS control group (**Table S2**). The standard curve of viral load using the qPCR method is shown in **Figure S2**. CyHV-2 loads among various tissues were significantly different (**Figure 2**). The viral loads in the kidney, spleen, midgut, hindgut, and gill were 4.4 × 10^5^, 3.5 × 10^5^, 3.0 × 10^5^, 1.6 × 10^5^, and 1.3 × 10^5^ copies ng^-1^ DNA, respectively. Less than 1.0 × 10^2^ copies ng^-1^ DNA were detected in the liver, foregut, muscle, and brain. Thus, different organs had different viral loads.

### 3.2 CyHV-2 infection changed gut microbiome

#### 3.2.1 OTU statistics and α-diversity analysis

We examined the microbiome in the midgut of infected and uninfected fish. The raw data about the microbiome and metabolites have been deposited in the NCBI (https://www.ncbi.nlm.nih.gov/sra/PRJNA655390), and the accession number is PRJNA655390. The abundance statistics of OTUs are shown in **Table S3**. Chao1 richness estimators in control and CyHV-2-infection groups were 926.60 and 985.54, respectively. The Simpson index in the CyHV-2-infection group was higher than that in the control group, and the Shannon index was lower. The less Simpson index, the higher the bacterial community diversity, while the Simpson index is inverse. The present results suggested that there was no significant difference in bacterial diversity between CyHV-2-infection and control groups (**Table S4**). Following the microbiota diversity analysis, we classified bacteria in fish midguts. A total of 383 OTUs were shared by the control and CyHV-2-infection groups. A total of 264 OTUs in control and 273 OTUs in the CyHV-2-infection group were unique, respectively (**Figure S3**).

#### 3.2.2 Microbiota composition

**Figure 3:**
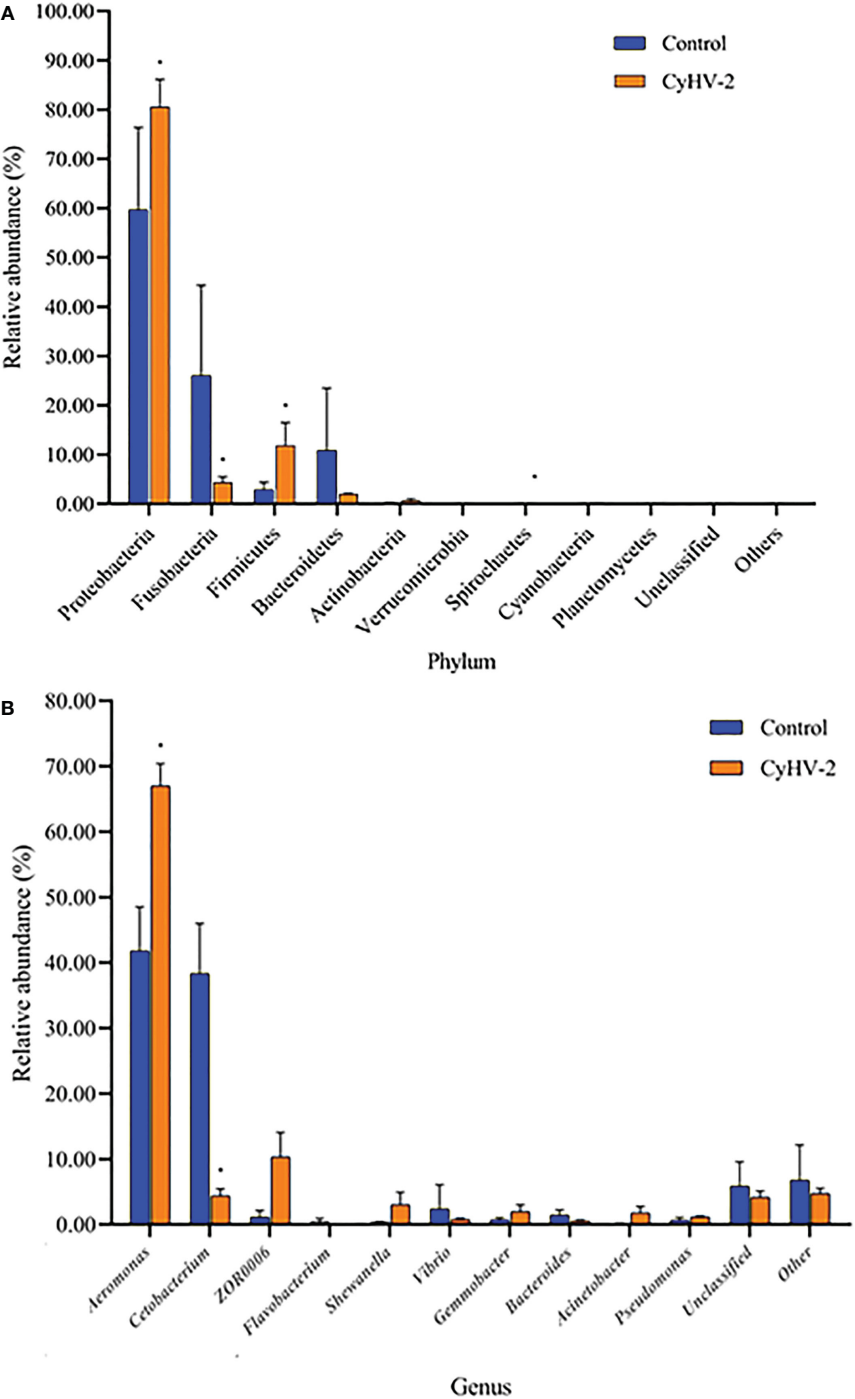
The gut microbiome composition individual profiles at the phylum **(A)** and genus **(B)** levels in the control and CyHV-2-infection groups. ^*^ means a significant difference between the two groups (*p*<0.05).

At the phylum level, the microbiota in both control and CyHV-2-infection groups were dominated by Proteobacteria, Fusobacteria, Firmicutes, and Bacteroidetes (**Figure 3A**). Notably, compared to the control group, the relative abundance of Proteobacteria and Firmicutes in the CyHV-2-infection group was significantly increased, while the abundance of Fusobacteria decreased (*p* < 0.05). At the genus level, the gut microbiota in both control and CyHV-2-infection groups were dominated by *Aeromonas*, *Cetobacterium*, ZOR0006, *Flavobacterium*, *Shewanella*, *Vibrio*, *Gemmobacter*, *Bacteroides*, *Acinetobacter*, and *Pseudomonas*. Compared to the control group, the relative abundance of *Aeromonas* in the CyHV-2-infection group increased from 41.81% to 67.00%, while *Cetobacterium* decreased from 38.36% to 4.41% (**Figure 3B**). Multivariate statistical analysis–Principal Co-ordinates Analysis PCoA was used to reveal the difference in midgut microbiome patterns that responded to CyHV-2 infection. The gut microbiota in the CyHV-2-infection group was clearly separated from the control group, with 69.12% and 22.65% variation explained by PC1 and PC2 at the genus level, respectively (**Figure S4A**). The result from hierarchical clustering analysis with unweighted pair-group method with arithmetic means UPGMA was consistent with the PCoA plot, i.e., all OTUs in both control and CyHV-2-infection groups were clustered in their own cluster except sample 2 by CyHV-2 infection (**Figure S4B**). Additionally, the analysis of similarities ANOSIM analysis showed that the R-value between the control and CyHV-2-infection groups was 0.63, illustrating that the middle difference between the two groups was found (**Figure S4C**). These differences of midgut microbiome patterns in different groups can be directly attributed to the relative abundance changes of dominant species. Therefore, CyHV-2 infection changed the abundance of different classes of bacteria in the midgut of fish.

**Figure 4:**
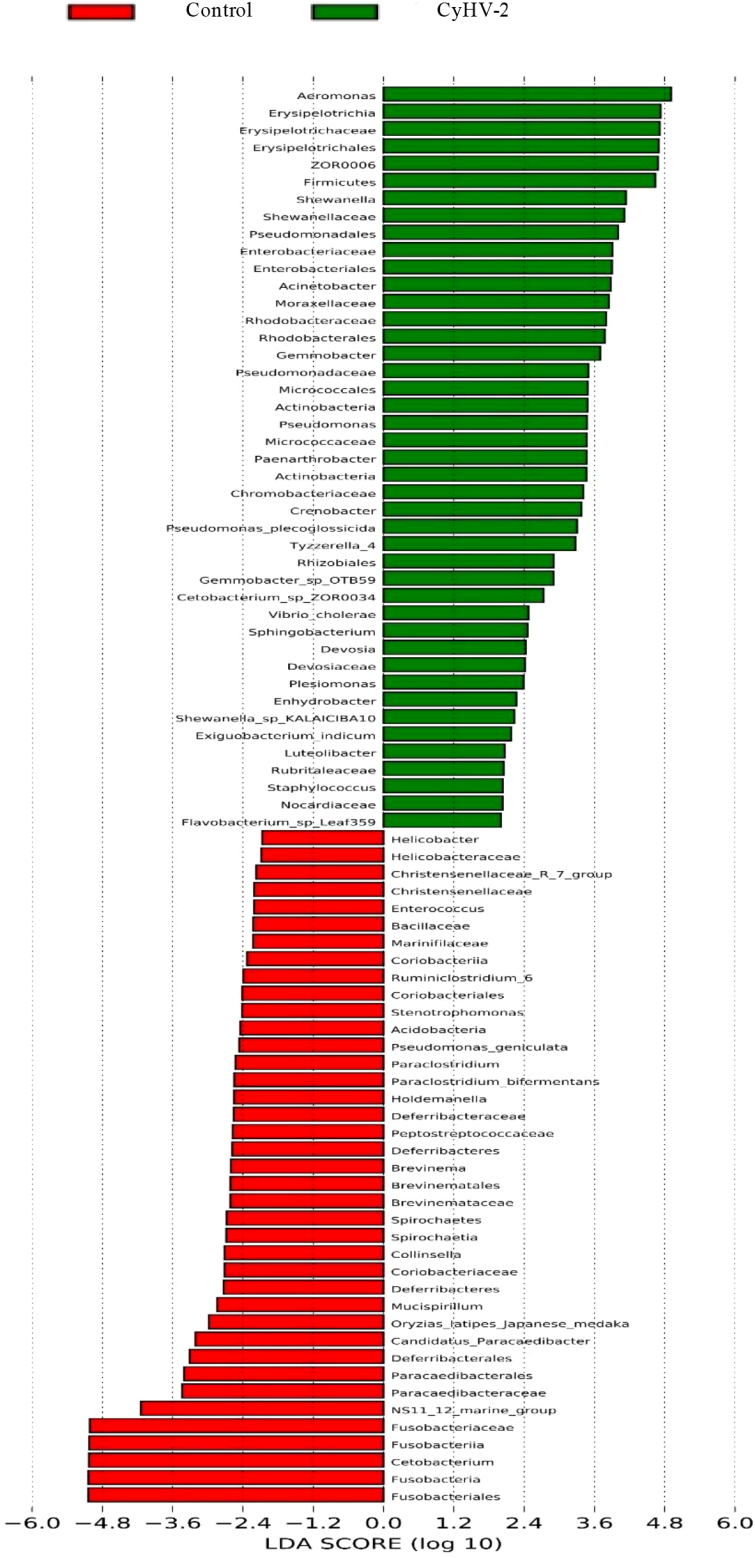
Linear discriminant analysis coupled with effect size measurements to identify the most deferentially abundant taxon between control and CyHV-2 infection groups.

**Figure 5:**
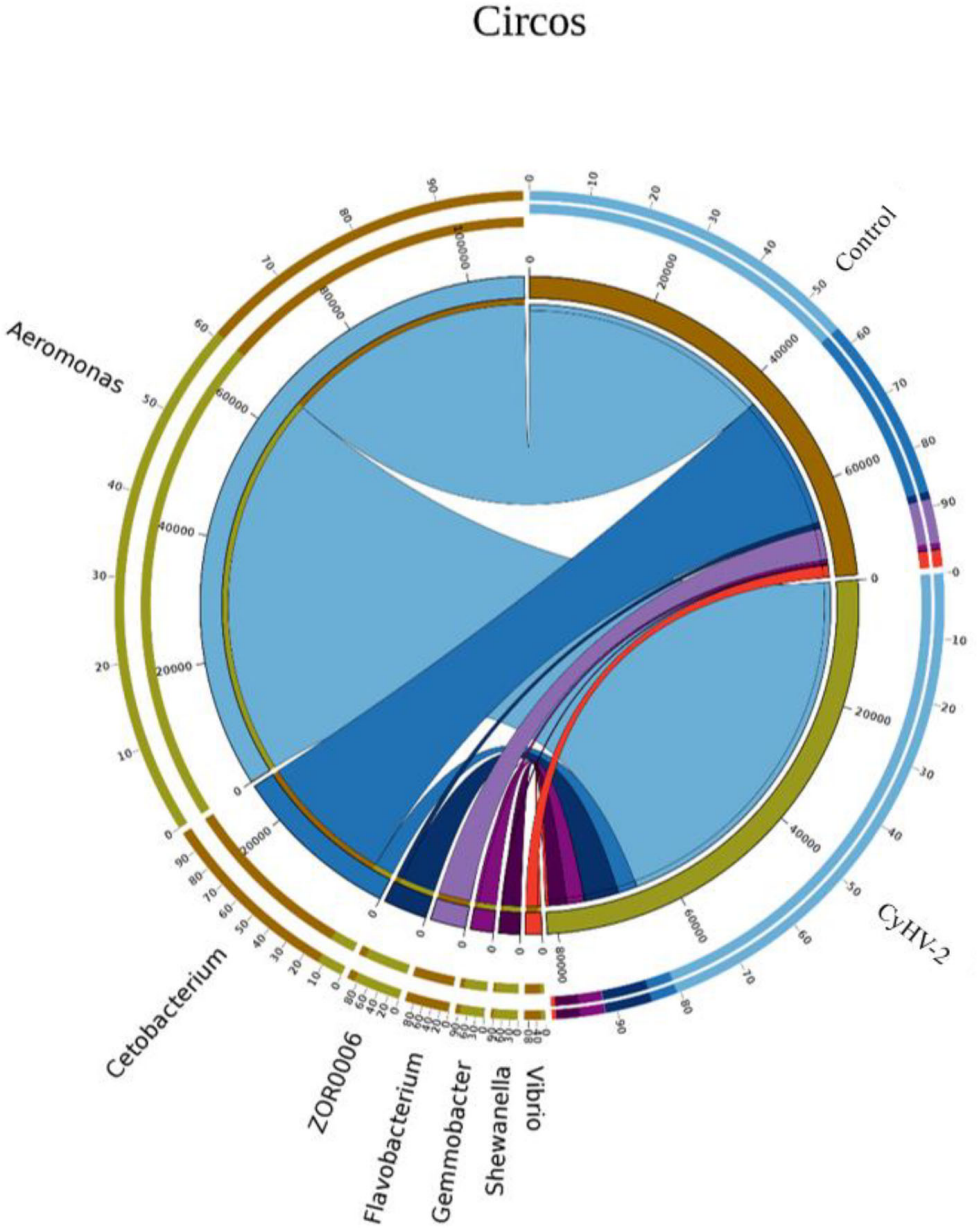
The relationship between the gut microbial composition and experimental treatments at the genus level by Circos based on canonical correspondence analysis (CCA).

LEfSe and CCA analysis showed that the specific main species in each group could be found. The most relevant species in the midgut-related CyHV-2 infection was *Aeromonas*. In the control group, *Fusobacteria* and *Cetobacterium* among different individuals were donated (**Figure 4**). *Aeromonas* presented a closely positive association with the viral infection (**Figure 5**). *Aeromonas* accounted for 41.81% in the control group and 67.00% in the CyHV-2-infection group (**Figure 3B**). A time-dependent abundance of *Aeromonas* in the midgut of gibel carp post-CyHV-2 infection by the culture method increased from 3.8 × 10^4^ CFU g^-1^ midgut at 0 dpi to 6.4 × 10^5^ CFU g^-1^ midgut at 1 dpi, 2.8 × 10^6^ CFU g^-1^ midgut at 4 dpi, and 7.6 × 10^7^ CFU g^-1^ midgut at 5 dpi, respectively. The abundance of *Aeromonas* at 5 dpi increased almost 1,000 times compared to 0 hpi, which was in accordance with the microbiome analysis by high-throughput sequencing. There was no significant difference in *Aeromonas* (less than 8 CFU ml^-1^ water) in culture water between the two groups during the whole challenge test. These results illustrated that CyHV-2 infection increased the relative abundance of *Aeromonas* and decreased *Cetobacterium.*

#### 3.2.3 Functional prediction of gut microbiota through PICRUSt

A total of 35 KEGG functions of all the midgut microbiota from two groups were annotated through PICRUSt. The major functions were donated into three categories, namely, metabolism, genetic information processing, and environmental information processing. The functions regulated by the top 10 gut microbiota were membrane transport, carbohydrate metabolism, amino acid metabolism, replication and repair, energy metabolism, translation, metabolism of cofactors and vitamins, nucleotide metabolism, cell motility, and lipid metabolism (**Figure S5A**). The different microbiota between the control and CyHV-2-infection groups were annotated into 16 functions (**Figure S5B**).

### 3.3 CyHV-2 infection altered metabolite profiles in fish gut

#### 3.3.1 Basic analysis of midgut metabolites from two groups

**Figure 6:**
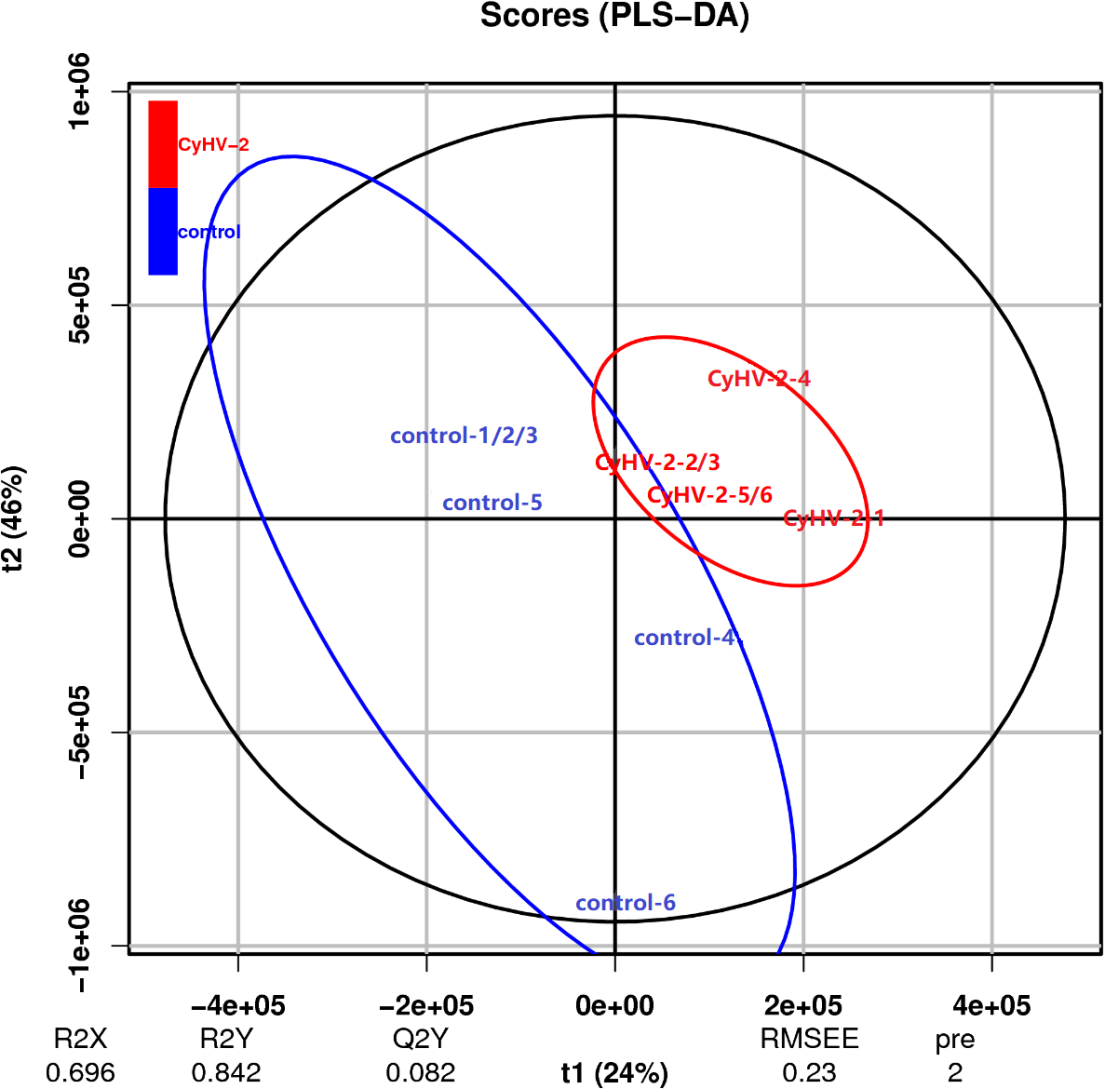
Scatter plot of partial least squares discriminant analysis for metabolite profiles from control and CyHV-2-infection groups. Each group was conducted in six replicates and named control-1/2/3/4/5/6 and CyHV-2-1/2/3/4/5/6. The distance of each point represents their correlation.

The PLS-DA model was conducted on the NMR data sets of all individuals, and the first two primary components PC1 and PC2 were selected to identify the discrimination between the control and CyHV-2-infection groups. Results showed that the metabolite profiles between the two groups were separated with 24% and 46% variation explained by PC1 and PC2, respectively (**Figure 6**).

#### 3.3.2 Different metabolites between two groups

Compared to the control, the concentrations of the main 24 metabolites were significantly changed in the CyHV-2-infection group. Among them, the concentrations of 8 metabolites (norizalpinin, D-maltose, (dibromomethylene)-17beta-hydroxy-androst-4-en-3-one propiote, deoxy-D-altro-heptulose 7-phosphate, ellagic acid, alanyl-valine, N-a-acetyl-L-arginine, and phosphophosphite) increased and 16 metabolites decreased significantly (**Table 1**). These metabolites are related to various metabolism pathways, such as the digestive system (p-cresol, D-glucose, and D-maltose), amino acid metabolism (2-oxo-4-methylthiobutanoic acid, phenylpyruvic acid, hydroxyphenyllactic acid, ketoleucine, O-phospho-L-serine, picolinic acid, 2-oxoarginine, and 2-oxoadipate), metabolism of other amino acids (L-selenocysteine), carbohydrate metabolism (D-maltose), lipid metabolism (phytosphingosine), xenobiotics biodegradation, and metabolism (cyclophosphamide).

**Table 1:** Differences in the metabolite profiles between the control and Cyprinid herpesvirus 2 (CyHV-2)-infection groups .

Metabolites	Class	Fold change	*P*-value	VIP
o-Cresol	Phenols	<0.01	<0.01	3.71
Phenylpyruvic acid	Benzene and substituted derivatives	0.05	<0.01	11.11
Indoleacetaldehyde	Indoles and derivatives	0.51	0.04	2.32
2-Oxo-4-methylthiobutanoic acid	Fatty acyls	0.02	<0.01	13.84
D-Lactic acid	Hydroxy acids and derivatives	0.53	<0.01	20.20
Succinic anhydride	Oxolanes	0.26	<0.01	2.00
8,11,14-Eicosatrienoic acid	Fatty acyls	0.56	0.02	6.95
Ketoleucine	Keto acids and derivatives	0.18	0.02	19.90
Iloprost	Fatty acyls	0.48	<0.01	3.26
Norizalpinin	Flavonoids	3.37	0.02	2.85
L-Selenocysteine	Carboxylic acids and derivatives	0.20	0.02	2.12
Convincing	–	0.51	0.01	3.37
6-(Dibromomethylene)-17beta-hydroxy-androst-4-en-3-one propiote	–	2.19	0.02	7.72
1-Deoxy-D-altro-heptulose 7-phosphate	–	36.00	<0.01	3.31
Ellagic acid	Tannins	6.43	<0.01	2.90
Picolinic acid	Pyridines and derivatives	0.43	0.02	4.64
D-Maltose	Organooxygen compounds	4.29	0.04	2.33
2,3,4,5-Tetrahydropiperidine-2-carboxylate	–	0.40	<0.01	4.85
Alanyl-Valine	–	3.37	0.04	3.58
4-Coumaroyl-2-hydroxyputrescine	Cinnamic acids and derivatives	0.07	<0.01	3.19
N-a-Acetyl-L-arginine	Carboxylic acids and derivatives	4.59	<0.01	8.86
2-Oxoarginine	Keto acids and derivatives	0.25	<0.01	7.28
Phosphophosphite	–	2.76	0.02	2.29
Nitrosylsulfuric acid	Non-metal oxoanionic compounds	0.31	<0.01	2.84

#### 3.3.3 Function annotation of metabolites

**Figure 7:**
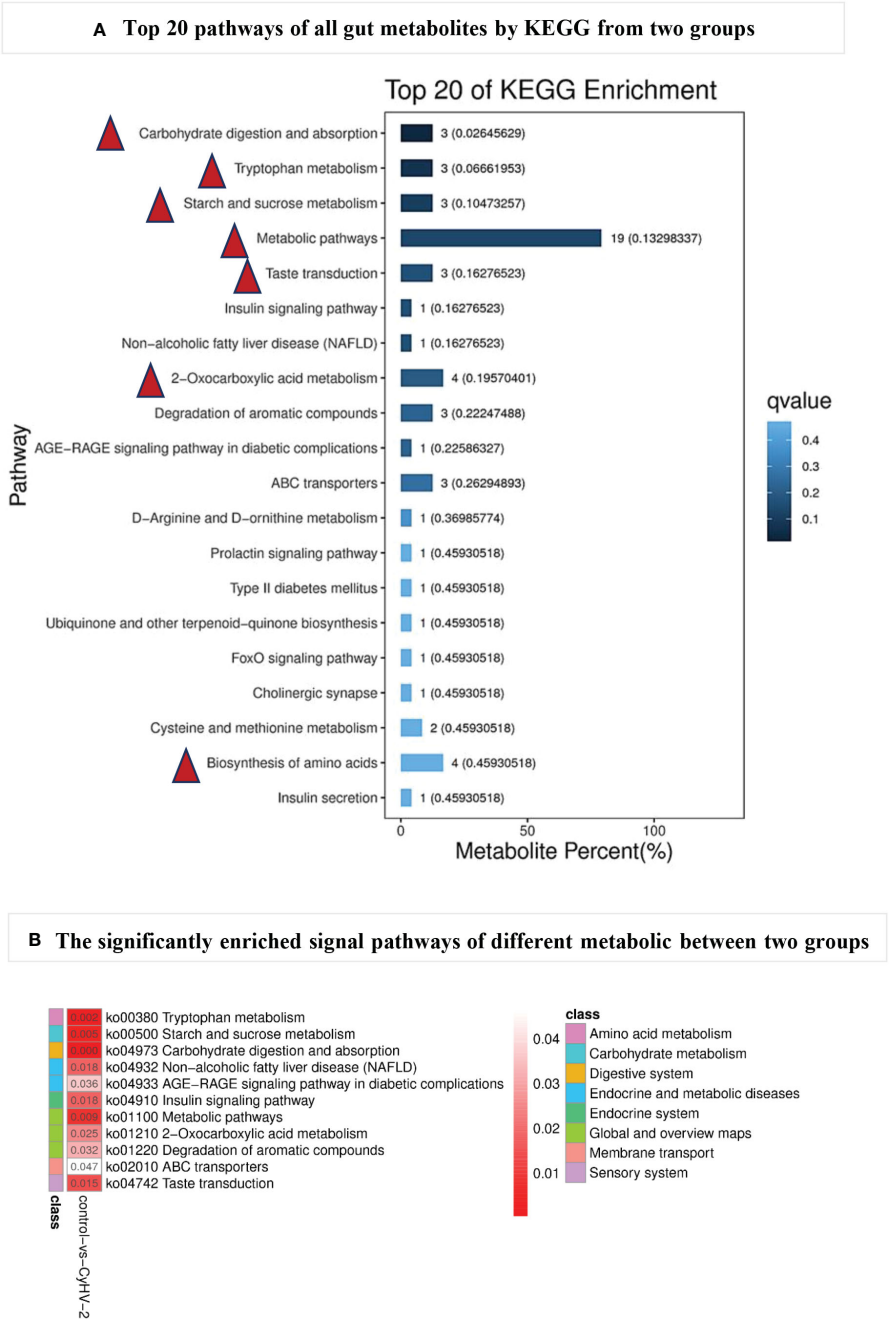
The top 20 pathways **(A)** of all gut metabolites from control and CyHV-2-infection groups through KEGG annotation and enriched signal pathways **(B)** with significant changes between two groups. The most top pathways of all gut metabolites are marked with a red triangle in **(A)** and the most significantly different pathways with a red arrow in **(B)** The colors and numbers in panel B represent the significance of metabolic pathways. The higher significance, the smaller the number and the darker the color.

All of the metabolites from both control and CyHV-2-infection groups were enriched, and the top 20 KEGG pathways are shown in **Figure 7A**. The first seven pathways were metabolic pathways, 2-oxocarboxilic acid metabolism, carbohydrate digestion and absorption, Trp metabolism, starch and sucrose metabolism, taste transduction, and biosynthesis of amino acids (**Figure 7A**). Different metabolites between the two groups were enriched in 11 signaling pathways (*p* < 0.05) (**Figure 7B**). Among them, the most obviously different signaling pathways were Trp metabolism, starch and sucrose metabolism, carbohydrate digestion and absorption, metabolic pathways, and taste transduction (**Figure 7**). Accordingly, different metabolites were mainly annotated to the digestive system, amino acid metabolism, carbohydrate metabolism, and lipid metabolism (**Table 2**). Taken together with metabolites and their related signaling pathways, we found that five metabolites were involved in these significantly different signaling pathways, including picolinic acid (PLA), 2-oxoadipate, and indoleacetaldehyde participated in Trp metabolism, phytosphingosine entangled in non-alcoholic fatty liver disease, D-maltose and D-glucose annotated in carbohydrate digestion and absorption, starch and sucrose metabolism, taste transduction, and ATP-binding cassette ABC transporters. Therefore, CyHV-2 infection changed midgut metabolites significantly, further affecting metabolisms, such as Trp metabolism.

**Table 2:** The correlation between some differentially specific microbes and metabolites.

Metabolites	Bacteria	Cor value	*P*-value	Class	KEGG annotation
PC	*Aeromonas*	-0.98	0.0006	Glycerophospholipids	Cancers
Glycyrrhetinic acid		0.97	0.001	Prenol lipids	Unknown
Sedoheptulose 1-phosphate		0.98	0.0008	Organooxygen compounds	Unknown
1-kestose		-0.98	0.0008	Organooxygen compounds	Unknown
Donepezil		0.98	0.0004	Piperidines	Unknown
8-iso-15-keto-PGE2		-0.98	0.0002	Fatty acyls	Unknown
2-oxoarginine		0.97	0.05	Keto acids and derivatives	Amino acid metabolism
Indoleacetaldehyde		-0.97	0.0007	Indoles and derivatives	Amino acid metabolism
Adenosine monophosphate	*Cetobacterium*	0.99	0.0002	Organooxygen compounds	Aging
LysoPC(24:0)		0.99	0.0001	Glycerophospholipids	Cancers
4-ketocyclophosphamide		0.98	0.0008	Organonitrogen compounds	Xenobiotics
D-glucono		0.98	0.0008	Organooxygen compounds	Carbohydrate metabolism
cis-aconitate		0.97	0.0009	Carboxylic acids and derivatives	Carbohydrate metabolism
cis-4-Carboxymethylenebut-2-en-4-olide		0.98	0.0007	Dihydrofurans	Global and overview maps
4-Pyridoxate		1.00	0.0000	Pyridines and derivatives	Metabolism of cofactors and vitamins
4-Hydroxybenzaldehyde		0.99	0.0002	Organooxygen compounds	Global and overview maps
Pyrrolidonecarboxylic acid		0.97	0.0013	Carboxylic acids and derivatives	Metabolism of other amino acids
Indoleacetaldehyde		0.99	0.0003	Indoles and derivatives	Amino acid metabolism
13-oxoODE		0.97	0.0011	Fatty acyls	Lipid metabolism
L-Cysteine		0.99	0.0003	Carboxylic acids and derivatives	Amino acid metabolism
Ketoleucine		0.98	0.0008	Keto acids and derivatives	Amino acid metabolism
4-hydroxyphenylpyruvic acid		0.99	0.0001	Benzene and substituted derivatives	Amino acid metabolism
Deoxyribose 5-phosphate		0.99	0.0001	Organooxygen compounds	Carbohydrate metabolism
stearoyl sphingomyelin		0.98	0.0007	Sphingolipids	Unknown
Phytosphingosine		-0.97	0.05	Organonitrogen compounds	Lipid metabolism
2-oxoarginine		0.97	0.003	Keto acids and derivatives	Amino acid metabolism
PC(22:6(4Z,7Z,10Z,13Z,16Z,19Z)	*Flavobacterium*	0.97	0.0011	Glycerophospholipids	Cancers
3’,5’-cyclic GMP		-0.98	0.0007	Purine nucleotides	Cellular community—eukaryotes
Squalene		-0.98	0.0006	Prenol lipids	Lipid metabolism
Sedoheptulose 7-phosphate	*Shewanella*	0.98	0.0006	Organic oxygen compounds	Carbohydrate metabolism
Palmitic acid		-0.97	0.0010	Fatty acyls	Lipid metabolism
2-amino-4-hydroxy-6-(D-erythro-1,2,3-trihydroxypropyl)-7,8-dihydropteridine		-0.97	0.0012	Pteridines and derivatives	Metabolism of cofactors and vitamins
Deoxyadenosine	*Vibrio*	0.99	0.0002	Purine nucleosides	Nucleotide metabolism
cis-aconitate		0.97	0.0010	Carboxylic acids and derivatives	Carbohydrate metabolism
6-methylthiopurine5’-monophosphate ribonucleotide	*Gemmobacter*	0.97	0.0011	Purine nucleotides	Xenobiotic biodegradation and metabolism
sn-glycerol 3-phosphate	*Bacteroides*	0.98	0.0009	Glycerophospholipids	Cancers
2,5-furandicarboxylate		-0.97	0.0011	Furans	Global and overview maps
GMP		-0.98	0.0005	Purine nucleotides	Nucleotide metabolism
Triethanolamine	*Acinetobacter*	-0.97	0.0013	Amines	Lipid metabolism
6-methylthiopurine5’-monophosphate ribonucleotide		0.99	0.0002	Purine nucleotides	Xenobiotic biodegradation and metabolism

### 3.4 Verification of the correlation between AhR and CyHV-2 infection

#### 3.4.1 CyHV-2 infection decreased the concentration of AhR in the midgut

**Figure 8:**
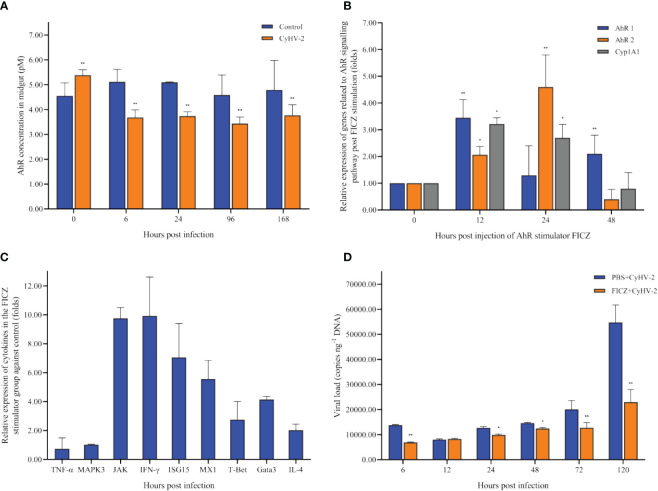
Time-dependent changes of the aryl hydrocarbon receptor (AhR) concentration in the midgut of fish post-CyHV-2 infection **(A)**, the effect of 6-Formylindolo[3,2-b]carbazole (FICZ) on the AhR signaling pathway **(B)**, the effect of FICZ on the transcription of cytokines **(C)**, and effect of FICZ on the viral replication *in vivo* **(D)**. All measurements are done in the midgut. ^*^ means a significant difference between two groups at a *p*-value from 0.01 to 0.05. ^**^ means a *p*-value less than 0.01.

In the control group, there was no significant difference on the concentration of AhR among 0, 6, 24, 72, and 120 hpi. However, in the CyHV-2-infection group, the concentration decreased from 5.38 pM (0 hpi) to 3.77 pM (120 hpi) (**Figure 8A**).

#### 3.4.2 *AhR* expression affected the transcription of cytokines

The optimal FICZ injection (I.P.) was 1 μg fish^-1^ for 12 hpi (**Figure 8B**). At 12 hpi, the highest expression of *AhR1* and *Cyp1A1* was observed, and *AhR2* upregulated significantly (**Figure 8B**). The transcriptional levels of cytokines *JAK*, *IFN-γ*, *ISG15*, *MX1*, *IL-4*, *Gata3*, and *T-Bet1* were upregulated significantly by 9.75-fold, 9.92-fold, 7.05-fold, 5.56-fold, 2.03-fold, 4.14-fold, and 2.75-fold, respectively. However, the mRNA expression of genes *TNF-α* and *MAPK3* was not regulated significantly (**Figure 8C**).

#### 3.4.3 Higher *AhR* expression reduced the cumulative mortality of fish and inhibited CyHV-2 replication *in vivo*

Compared to the ‘PBS+CyHV-2’ infection group, the CM of fish post-CyHV-2 infection in the ‘FICZ+CyHV-2’ group was much lower. CM in the ‘FICZ+CyHV-2’ group at the viral concentrations of 2.4 × 10^7^ and 9.7×10^6^ copies μl^-1^ was 41.7% and 33.3%, respectively. They were 83.8% and 58.3% in the ‘PBS+CyHV-2’ group. The fish in the ‘FICZ+CyHV-2’ group died later than that in the ‘PBS+CyHV-2’ group. No death was observed in PBS single and FICZ single injection groups (**Table 3**).

**Table 3:** Effect of aryl hydrocarbon receptor (AhR) expression on the cumulative mortality of fish post-CyHV-2 infection.

Groups	Days post infection														Cumulative mortality (%) (mean ± SD)
	1	2	3	4	5	6	7	8	9	10	11	12	13	14	
PBS	0	0	0	0	0	0	0	0	0	0	0	0	0	0	0
PBS+CyHV-2 (2.4×10^7^ copies μl^-1^)	0	0	1	0	0	0	0	2	2	0	1	1	0	0	83.8 ± 7.2
PBS+CyHV-2 (2.4×10^7^ copies μl^-1^)	0	0	0	0	1	0	0	2	0	0	1	1	1	0	
PBS+CyHV-2 (2.4×10^7^ copies μl^-1^)	0	1	1	0	0	0	0	2	1	0	1	1	0	0	
PBS+CyHV-2 (9.7×10^6^ copies μl^-1^)	0	0	0	0	0	0	0	0	1	1	1	0	0	1	58.3 ± 7.2
PBS+CyHV-2 (9.7×10^6^ copies μl^-1^)	0	0	2	0	0	0	0	0	0	0	0	2	1	0	
PBS+CyHV-2 (9.7×10^6^ copies μl^-1^)	0	0	1	1	0	0	0	1	0	1	0	1	0	0	
FICZ	0	0	0	0	0	0	0	0	0	0	0	0	0	0	0
FICZ+CyHV-2 (2.4×10^7^ copies μl^-1^)	0	0	1	2	0	0	0	0	0	0	0	1	0	0	41.7 ± 7.2
FICZ+CyHV-2 (2.4×10^7^ copies μl^-1^)	0	0	0	0	0	0	0	2	0	0	0	1	0	0	
FICZ+CyHV-2 (2.4×10^7^ copies μl^-1^)	0	0	0	0	1	0	0	1	0	1	0	0	0	0	
FICZ+CyHV-2 (9.7×10^6^ copies μl^-1^)	0	0	0	0	0	0	0	1	0	0	0	0	0	1	33.3 ± 7.2
FICZ+CyHV-2 (9.7×10^6^ copies μl^-1^)	0	0	1	0	0	0	0	0	1	0	1	0	0	0	
FICZ+CyHV-2 (9.7×10^6^ copies μl^-1^)	0	0	0	0	0	1	0	1	0	0	0	0	0	1	

FICZ, 6-formylindolo [3,2-b] carbazole, a tryptophan photoproduct postulated as a candidate physiological ligand of AhR.

Compared to the ‘PBS+CyHV-2’-infection group, the viral replication in the ‘FICZ+CyHV-2’ group was slower, suggesting that higher *AhR* expression inhibited CyHV-2 replication *in vivo*. The viral load in the ‘FICZ+CyHV-2’ group at 6 hpi (6.9 × 10^3^ copies ng^-1^ DNA) and 120 hpi (2.3 × 10^4^ copies ng^-1^ DNA) was lower than that in the ‘PBS+CyHV-2’ group, which were 1.4 × 10^4^ copies ng^-1^ DNA at 6 hpi and 5.5 × 10^4^ copies ng^-1^ DNA at 120 hpi, respectively (**Figure 8D**). These findings and in-depth verification suggested that Trp metabolism is one of the cross-talk ways between a viral infection and a host.

### 3.5 Correlation between the altered microbiota and metabolites

**Figure 9:**
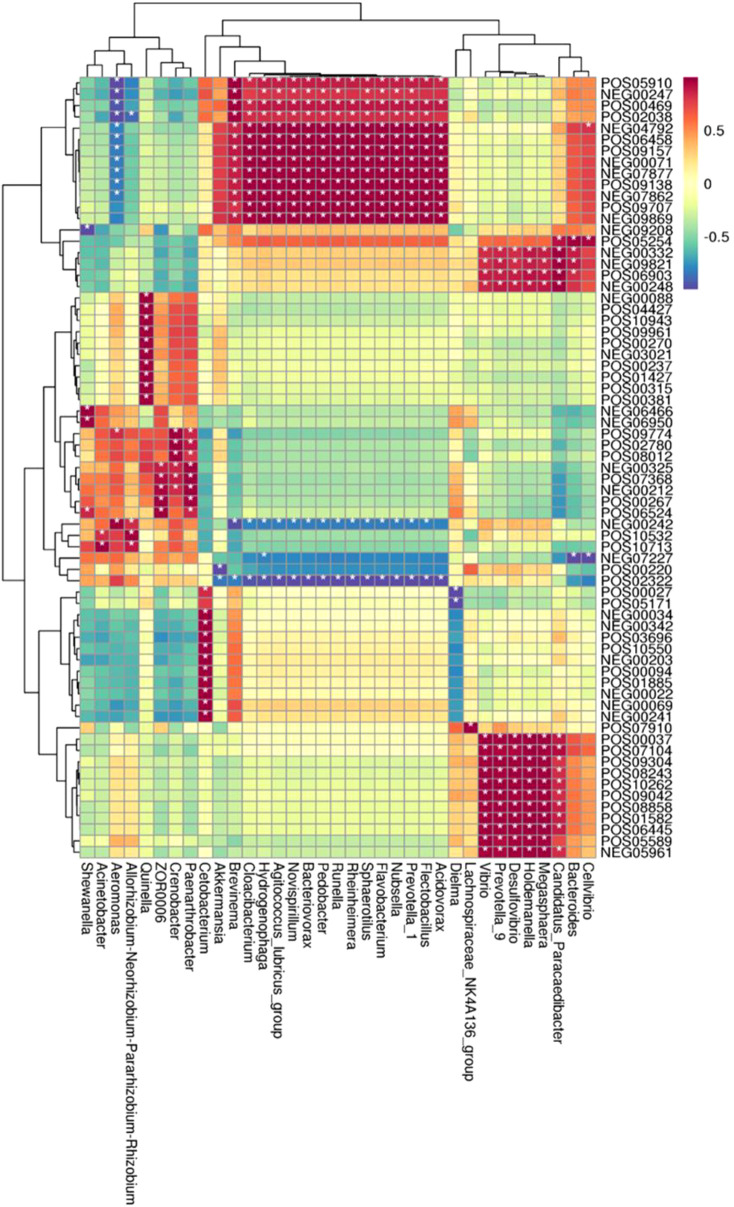
Heatmap of the correlation between gut metabolites and the microbiota. The horizontal axis represents bacterial species, and the vertical axis represents the metabolites. Grid means the correlation index. The color from white to red means a positive correlation from weak to strong, and white to blue means a negative correlation. *, significant correlation with a *p*-value less than 0.05; **, highly significant correlation with a *p*-value less than 0.01.

**Figure 10:**
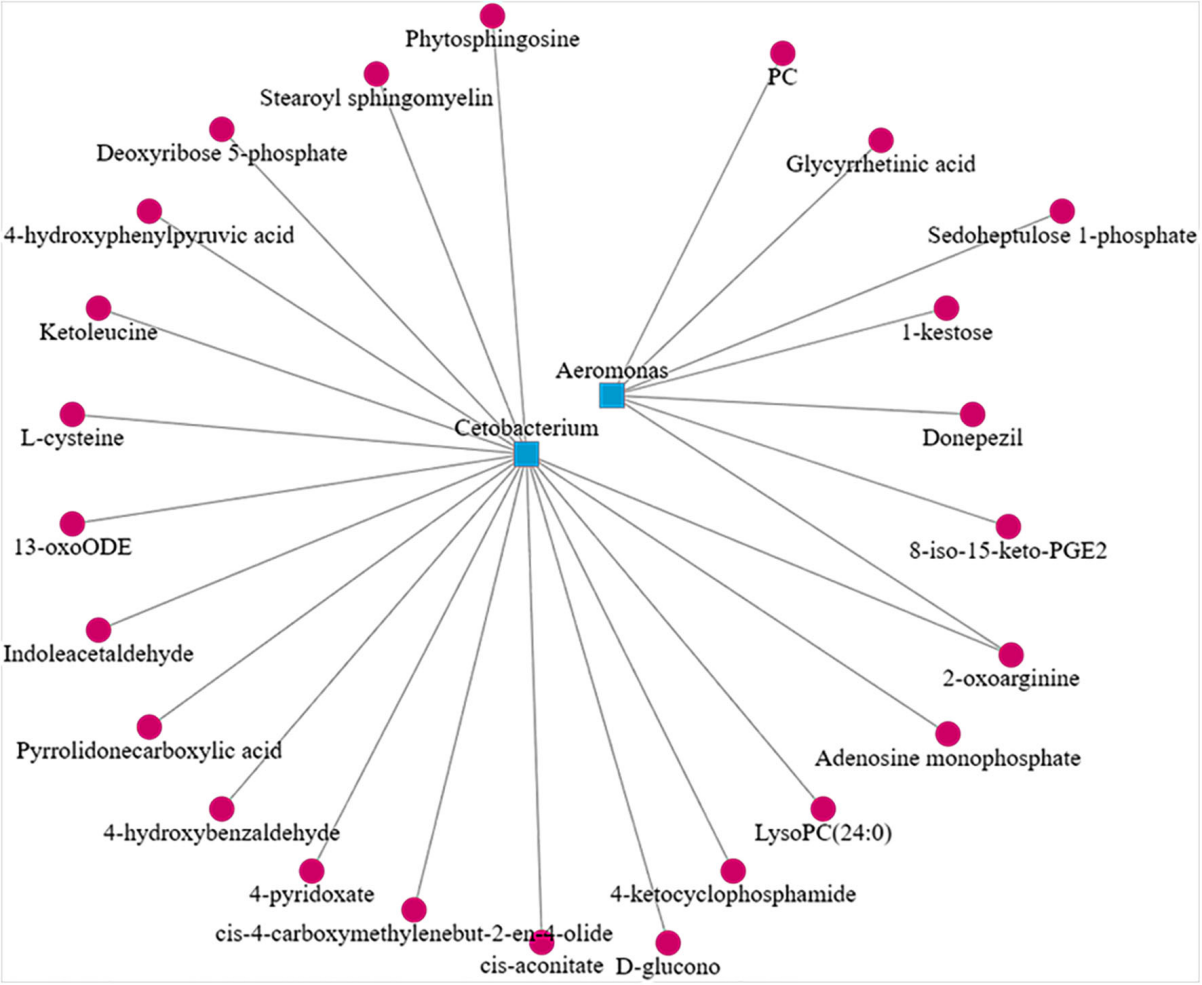
Scatter plot showing the correlation between *Aeromonas*, *Cetobacterium*, and significant different metabolites from the control and CyHV-2-infection groups.

In the CyHV-2-infection group, the relative abundance of *Aeromonas* significantly increased, while *Cetobacterium* decreased. We generated a scatter plot using Pearson correlation coefficient analysis to investigate the potential association between metabolites and bacterial composition. The heatmap of the correlation between gut metabolites and microbiota is shown in **Figure 9**. The apparent correlations were identified between the perturbed bacteria genus and altered metabolites (Mantel test, *r* = 0.89) (**Figure 10** and **Table 2**). For example, the OTUs assigned to *Aeromonas* were positively correlated with glycyrrhetinic acid, sedoheptulose 1-phosphate, donepezil, and 2-oxoarginine and negatively correlated with indoleacetaldehyde, 1-kestose, and 8-iso-15-keto-PGE2. OTUs belonging to *Cetobacterium* were positively correlated with indoleacetaldehyde, adenosine monophosphate, 4-ketocyclophosphamide, LysoPC (24:0), D-glucono, cis-aconitatecis-4-carboxymethylenebut-2-en-4-olide, 4-pyridoxate, 4-hydroxybenzaldehyde, pyrrolidonecarboxylic acid, 13-oxoODE, L-cysteine, ketoleucine, stearoyl sphingomyelin, deoxyribose 5-phosphate, phytosphingosine, 2-oxoarginine and 4-hydroxyphenylpyruvic acid (**Figure 10** and **Table 2**). In this study, the most noteworthy functional genes were involved in ABC transporters and glycerophospholipid metabolism related to membrane synthesis and gut integrity, followed by primary immunodeficiency (**Figure S5B**). Our data demonstrated that CyHV-2 infection changed both the gut microbiome and metabolome, and these two changes were correlated.

### 3.6 Alteration of AhR expression affected microbiota in the midgut of fish post CyHV-2 infection

**Figure 11:**
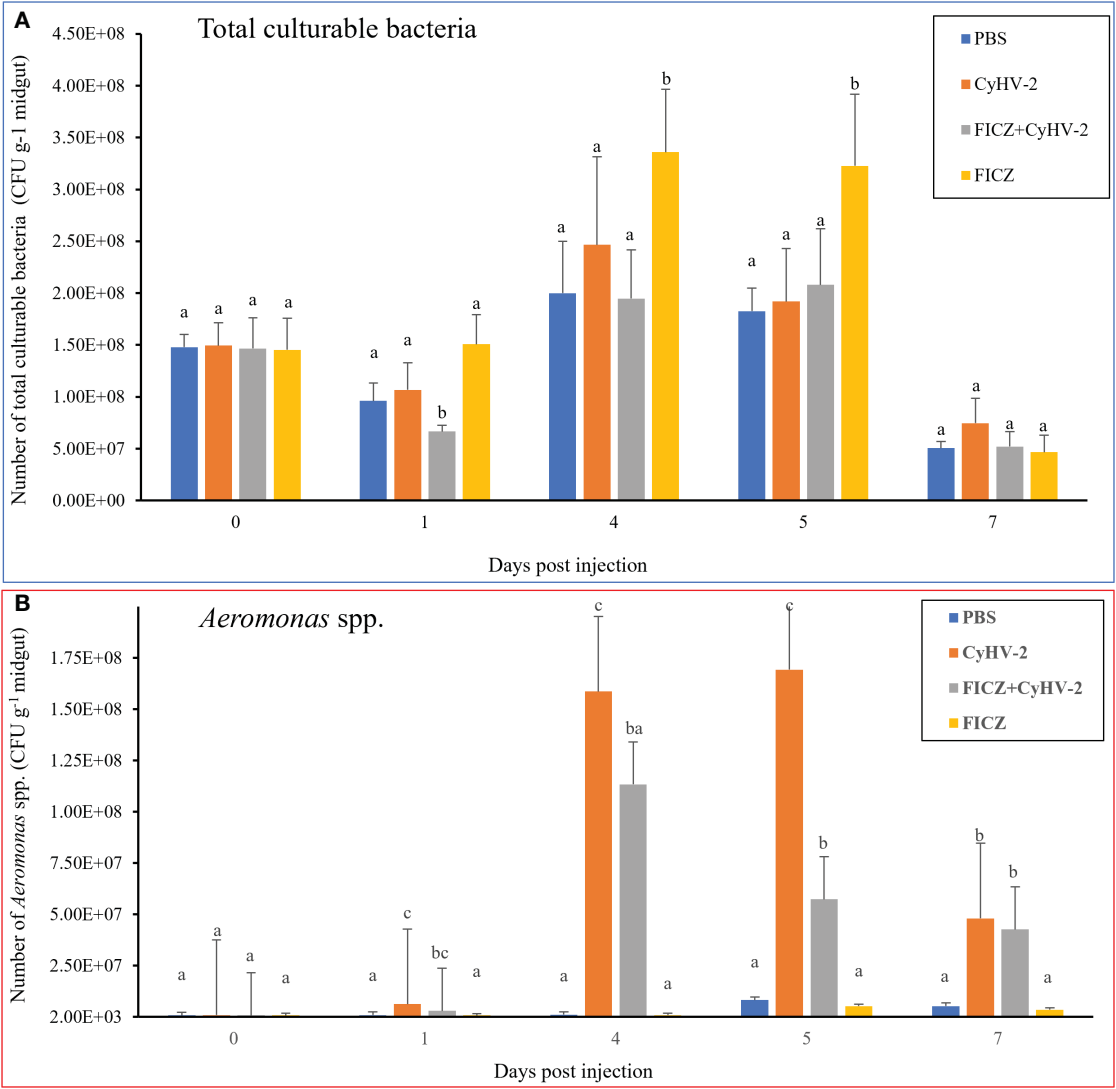
Effect of AhR expression on the number of total culturable bacteria and *Aeromonas* spp. in the midgut of fish post-CyHV-2 infection The data showing as 'mean±SE' is based on four biological replicates. The significant differences among different treatments are indicated by lowercase letters above each column (one-way ANOVA followed by the LSD test, P < 0.05) in **(A, B)**.

Compared to the PBS injection group, CyHV-2-infection group, and ‘FICZ+CyHV-2’ group, FICZ stimulation at 4 and 5 dpi increased the number of total culturable bacteria by approximately two folds significantly. There was no significant difference among the PBS injection group, CyHV-2-infection group, and ‘FICZ+CyHV-2’-infection group (**Figure 11A**). However, compared to PBS injection group and FICZ injection group, the number of *Aeromonas* spp. in the CyHV-2-infection group and ‘FICZ+CyHV-2’- infection group from 1 to 7 dpi increased significantly. There was still a significant difference between the CyHV-2-infection group and the ‘FICZ+CyHV-2’ infection group from 1 to 7 dpi. The number of *Aeromonas* spp. in the CyHV-2-infection group was significantly higher than that in the ‘FICZ+CyHV-2’ infection group from 1 to 7 dpi. FICZ injection could inhibit the replication of *Aeromonas* spp. in the midgut of fish post-CyHV-2 infection (**Figure 11B**).

## 4 Discussion

Gibel carp can be artificially infected by CyHV-2 through the ‘per-gill’ method. Like natural infection, the typical clinical syndromes of hemorrhagic gills and the swim bladder were observed post-CyHV-2 infection through the ‘per-gill’ method, and CyHV-2 could be quantified in different inner organs. The hemorrhage of gills is mainly observed during the transition from spring to summer and in autumn, similar to the epidemiological characteristics of the hematopoietic necrosis (HVHN) of goldfish infected with CyHV-2 (Jung and Miyazaki, 1995; Zhu et al., 2015). Until now, there is no effective method to control this disease, although some studies on a vaccine are undergoing in the lab (Takafumi and Yukio, 2015; Yan et al., 2020). Early prevention before disease outbreaks becomes more important to control this epidemic disease. Growing pieces of evidence have demonstrated that the gut microbiota plays an important role in health and diseases. The gut microbiota in fish can regulate the expression of 212 genes, of which some are related to the promotion of nutrient metabolism, stimulation of epithelial proliferation, and immunity. The absence of gut microbiota in fish may lead to epithelial cell dysfunction and weaker immune responses (Wang et al., 2018). Colonization by commensal in newly hatched zebrafish (*Denio rerio*) primes neutrophils and induces several genes encoding proinflammatory and antiviral mediators (Galindo-Villegas et al., 2012).

To our knowledge, this study was the first to address the relationship among CyHV-2 infection, gut microbiota, and metabolite profiles in the gibel carp. The present results showed (1) gut microbiota dysbiosis, especially the relative abundance increment of *Aeromonas* and decrement of *Cetobacterium*; (2) metabolome alternation regarding amino acid metabolism, carbohydrate metabolism, and lipid metabolism, especially Trp metabolism; (3) the strong correlation between gut microbiota and metabolite profiles; and (4) the interference of viral infection through modulating gut ecology. The predominant genera in the gibel carp were *Aeromonas*, *Cetobacterium*, *Flavobacterium*, *Shewanella*, *Vibrio*, *Gemmobacteria*, *Bacteroides*, *Acinetobacter*, and *Pseudomonas*. Accordingly, *Aeromonas*, *Cetobacterium*, *Pseudomonas*, *Acinetobacter*, and *Bacteroides* were reported as the predominant genera in freshwater fish (Li et al., 2017; Wang et al., 2018). However, there are some differences in genus composition; *Flavobacterium*, *Shewanella*, and *Vibiro* were found in our experimental fish, while these genera are mainly reported in marine fish (Onarheim et al., 1994; Blanch et al., 1997). It is supposed that this difference is mainly attributed to the culture environment, especially salinity, since our experimental fish was obtained from Yancheng and the culture sediment in the pond was closer to the mudflat (Chen et al., 2021). The culture farm in Yancheng is mainly located along the coastline with the longest and biggest mudflat in China. Consistent to the previous report (Zhang et al., 2016), the abundance of dominant species such as *Cetobacterium* increases along with higher salinity.

The microbiota depends on a functional equilibrium related to the host, environment, and dietary factors. The gut microbiome is responsible for maintaining gut metabolic homeostasis and coordinating immune responses (Kau et al., 2011; Shapira, 2016). A disturbance in the functional equilibrium can lead to changes in the microbial diversity and abundance of certain bacteria, which are beneficial or harmful to fish (Liu et al., 2013; Ling et al., 2014; Lukens et al., 2014). Thus, we used a laboratory infection model instead of naturally infected fish to study the effect of CyHV-2 infection on the gut microbiota and microbiome. After acclimation, the gut microbiota is more stable (Chen et al., 2021). In this study, the midgut of fish was chosen as the target organ to study the microbiome and metabolite changes post-CyHV-2 infection since the viral load in the midgut was higher than that in the foregut and hindgut (**Figure 2**). Accordingly, the microbial abundance and gene *gata*5 expression in the midgut of zebrafish are higher than that in the foregut and hindgut (Hu, 2017; Rong et al., 2017). The *gata*5 gene regulates gut mucus secretion and gut epithelial differentiation (Hu, 2017).

CyHV-2 infection changed the gut microbiota of fish. The abundance of genera *Aeromonas*, *Pseudomonas*, and *Flavobacterium* were significantly increased in the CyHV-2-infected fish. Particularly, the relative abundance of *Aeromonas* in the CyHV-2-infected fish increased significantly from 48.74% to 67.00%, consistent with reports in crucian carp *(C. carassius)* (Caruso et al., 2017). Accordingly, a time-dependent abundance of *Aeromonas* in the midgut of gibel carp increased along with the CyHV-2 infection progress. However, it is different from the previous report (Rong et al., 2017), which showed that *Plesiomonas* was highly abundant in CyHV-2-infected gibel carp and could be used as a microbial biomarker for CyHV-2 infection. In this study, no dominant *Plesiomonas* was detected in both uninfected and infected gibel carp, as in the previous report by Chen et al. (2021). This difference might be related to the environment salinity, water, and developmental stage, which have been widely reported to affect the fish gut microbiota (Wang et al., 2018). This study suggests that the abundance of *Aeromonas* could be attractively monitored during the disease development progress. *Aeromonas* are ubiquitous inhabitants of freshwater and estuary environments as potential etiological agents in diseases of fish, terrestrial animals, and humans. It can cause motile aeromonad septicemia with high mortality and severe economic losses (Ran et al., 2018).

The gut metabolic disorders were observed in the CyHV-2-infected fish. For instance, three digestive pathways related to metabolites of p-cresol, D-glucose, and D-maltose and eight amino acid metabolism–related pathways to the metabolites of 2-oxo-4-methylthiobutanoic acid, phenylpyruvic acid, hydroxyphenyllactic acid, ketoleucine, O-phospho-L-serine, PLA, 2-oxoarginine, and 2-oxoadipate were changed in the gut of CyHV-2-infected fish. PLA has broad and effective antimicrobial activity against both bacteria and fungi (Dieuleveux et al., 1998; Lavermicocca et al., 2003). PLA is a major metabolic biomarker for oxidative damage to the cerebral cortex, phenylketonuria, and alcohol-induced liver disease (Manna et al., 2011). More recently, PLA has been well documented to play a positive role in the progression of different cancers (Lavermicocca et al., 2003; Fong et al., 2011) as shown in this study. Glucose as a good carbon source for cofactor regeneration in Trp metabolism is consistent with the phenomenon that the glucose content increased, while the corresponding maltose content decreased significantly in this study. D-maltose and D-glucose are involved in the digestive system. Sugars are the primary carbon source for bacterial cells, and they play important roles in bacterial pathogenesis (Pacheco et al., 2012; Ng et al., 2013). Both pathogenic and commensal bacteria compete for carbon sources such as glucuronate, mannose, fucose, and ribose to colonize and proliferate in the gut (Lustri et al., 2017). Sugar availability influences the microbiota composition and expansion of bacterial pathogens (Lustri et al., 2017). The change of glucose concentration in the environment regulates the expression of the locus of enterocyte effacement and catabolic, and osmotic stress (Njoroge et al., 2012; Njoroge et al., 2013).

In this study, an apparent correlation between specific microbes and metabolites was observed. For instance, *Cetobacterium* OTUs are negatively correlated with phytosphingosine, which is involved in membrane synthesis. The concentration increment of phytosphingosine raises the permeability of endothelial cells and leads to the barrier function decline (Luther et al., 2013). Another interesting metabolite is 1-kestose, the smallest component of Fructooligosaccharide FOS in the probiotics from the human gut. These findings suggest that 1-kestose is a potential new prebiotic targeting drug (Tochio et al., 2018). It has been shown that the hepatocyte membrane has abundant glycyrrhetinic acid (GA) receptors (Shan et al., 2017). Some documents have authenticated that GA is combined with the GA receptors of hepatocyte membrane to enhance the targeted therapy of liver (Shan et al., 2017; Wang et al., 2018). A more interesting finding is that *Cetobacterium* OTUs are correlated with indoleacetaldehyde positively (Natividad et al., 2018). Both indoleacetaldehyde and PLA from this study were annotated to Trp metabolism. Actually, most indole derivatives are formed from Trp metabolism (Lamas et al., 2018). Indole substances are the endogenous ligands of AhR and thus participate in many important biological processes of the body, such as cell differentiation, apoptosis, and inflammation (Agus et al., 2018). Indole and Trp metabolites are a major source of endogenous AhR ligand precursors (Bjeldanes et al., 1991; Perdew and Babbs, 1991). It has been reported that the gut microbiota affects health and disease through the regulation of Trp metabolism and the activation of AhR (Lamas et al., 2016; Lamas et al., 2018; Agus et al., 2018; Natividad et al., 2018; Trikha and Lee, 2019). CARD9 was reported to impact colitis by altering the gut microbiota metabolism of Trp into AhR ligands (Lamas et al., 2016).

AhR is a member of the basic helix–loop–helix–(bHLH) superfamily of transcription factors, which are associated with cellular responses to environmental stimuli. Early AhR studies focused on understanding the role of AhR in mediating the toxicity and carcinogenesis properties of the prototypic ligand 2,3,7,8-tetrachlorodibenzo-p-dioxin (TCDD). Recently, AhR has been highly receptive to a wide array of endogenous and exogenous ligands. Its activation leads to a myriad of crucial host physiological functions, such as intestinal barrier function and immune cells, as well as intestinal homeostasis (Lamas et al., 2018). The byproducts of Trp photooxidation possess a high AhR-binding capacity, and are able to induce the expression of *Cyp*1A1 and other AhR target genes (Paine, 1976; Paine and Francis, 1980; Goerz et al., 1996). In fish, two kinds of AhR (AhR1 and AhR2) have been reported mostly (Prasch et al., 2003; Aluru et al., 2011). Thus, the AhR pathway related to Trp metabolism in this study was chosen to prove the effect of gut micro-ecology changes on the viral infection process through FICZ stimulation, which is a prime example of a Trp photoproduct and can agonistically stimulate AhR activity in as low as picomolar ranges (Jeong et al., 2012). The present results showed that 1) CyHV-2 infection reduced AhR concentration in the midgut (**Figure 8A**) and serum (data not shown), 2) higher *AhR* expression upregulated the mRNA expression of antiviral genes in the head kidney; 3) higher *AhR* expression reduced the relative abundance of *Aeromonas* spp. in the midgut of fish post-CyHV-2 infection; 4) higher *AhR* expression reduced the 14-day CM of fish post-CyHV-2 infection and inhibited viral replication *in vivo*. In mammals, AhR is known to express by different intestinal immune cells, such as intestinal epithelial cells (IECs), Th17 cells, innate lymphoid cells (ILCs), macrophages, and neutrophils. The AhR signaling pathway is pivotal to the differentiation and proliferation of T cells and the regulation of mucosal intestinal immune responses (Qiu et al., 2012; Goudot et al., 2017). Although the correlation between metabolite changes and CyHV-2 infection focusing on Trp metabolism was proven, further in-depth studies on the correlation among gut microbes, gut barrier disturbance, *Aeromonas* translocation, Trp metabolism, immunity, and antiviral infection need to be conducted. Especially, regulating Trp metabolism in the gut by diets including probiotics will attract more attention as reported in mammals (Whitfield-Cargile et al., 2016; Cervantes-Barragan et al., 2017). These actionable studies will probably provide an excellent immunotherapeutic intervention to control carp diseases and thus advance the sustainable culture.

## 5 Conclusion

This study found a significant difference in microbiota-regulated metabolites in the fish midgut post-CyHV-2 infection. Notably, the relative abundance of *Aeromonas* increased while *Cetobacterium* decreased. The different metabolites were annotated to the metabolism of the digestive system, amino acid, carbohydrate, lipid, and xenobiotic biodegradation, especially Trp metabolism. An in-depth analysis showed that some of these altered metabolites were highly correlated with the genera *Aeromonas* and *Cetobacterium*. Our results supported that CyHV-2 infection–induced changes in the midgut, bacterial community structure disturbed the metabolic functions of the gut microbiome, and the viral infection process could be interrupted through the modulation of gut microecology. These findings will provide a new therapeutic strategy to prevent disease and thus advance the sustainable culture.

## Data availability statement

The datasets presented in this study can be found in online repositories. The names of the repository/repositories and accession number(s) can be found below: National Center for Biotechnology Information (NCBI) BioProject, https://www.ncbi.nlm.nih.gov/bioproject/, PRJNA655390.

## Ethics statement

This study was conducted in accordance with the regulations for the administration of laboratory animals in Jiangsu province, China.

## Author contributions

PC and MZ performed project administration, original draft, editing, and funding acquisition. JL and XW help with data interpreting and writing. YZ and YC performed formal analysis, software, and visualization. TL performed conceptualization and data curation. LC performed conceptualization and investigation. ZZ provided fish. ZL and ZM performed methodology and software. GQ, ZGZ, and ZW performed conceptualization, funding acquisition, project administration, resources, supervision, validation and visualization, review and editing. All authors contributed to the article and approved the submitted version.
