# Supplementary material for: Cyprinid herpesvirus 2 infection changes microbiota and metabolites in the gibel carp (Carassius auratus gibelio) midgut
Source: Front Cell Infect Microbiol. 2023 Feb 2;12:1017165. doi: 10.3389/fcimb.2022.1017165 (PMC9933507; doi:10.3389/fcimb.2022.1017165)
Supplement: Supplementary file 1 [file DataSheet_1.doc]

**TA B L E S1** Primers used in this study

| **Genes** | **Forward primer sequence (5′→3′)** | **Reverse primer sequence (5′→3′)** |
| --- | --- | --- |
| Primers for CyHV-2 detection by PCR | GGACTTGCGAAGAGTTTGATTTCTAC | CCATAGTCACCATCGTCTCATC |
| Primers for CyHV-2 quantification by qPCR | CCCAGCAACATGTGCGACGG | CCGTARTGAGAGTTGGCGCA |
| V4 region amplification of bacterial 16S rRNA for microbiome analysis | CCTACGGGNGGCWGCAG | GGACTACHVGGGTATCTAAT |
| *AhR*1 | AGGGAAGATTGTTCTGGGCTA | CCGTCCTCATCATCCGTATG |
| *AhR*2 | CCAATTCCCAGTTCACCCTT | TCCGATTCGTCCACATCAAA |
| *Cyp1A1* | GATCCGTCAGTGGTGGTCTCAAAG | GCAGAGCGAAGGCACAGAGAAC |
| *IFN-γ* | CCCAGTCGTCCAGCACAAAGC | TTTGTCGTCTCCTGCGCTCTTTG |
| *MX1* | ATCTGGTGGATAAGGGAAC | CATCCTCTGTTAATGTGGC |
| *ISG15* | CGCACAAATGAACAATCACC | GGCATTACATGACCAAGTCTCA |
| *JAK* | CCATCCAGGACAGCAACATAACCC | AGATCCCTCGCTCTCACCAGATAC |
| *TNF-α* | CATTCCTACGGATGGCATTTACTT | CCTCAGGAATGTCAGTCTTGCAT |
| *MAPK3* | TGCTCAACTCCAAGGGCTACA | ACATGCGGTCCAATAAATCCA |
| *IL-4* | GTGAGTCTCCTGAACAGCCTGAAC | AGTGTGATGAGTTGTGGCGTCTTC |
| *Gata3* | ACACAATATCAACCGACCGCTCAC | TTGCTCTTCTTGGACTTGCTGGAC |
| *T-bet1* | CTTCACGAGAACCGATTCCGATGG | TCTGACAGATTCACAGCGTTTGGG |
| β-actin | GATGATGAAATTGCCGCACTG | ACCGACCATGACGCCCTGATGT |

CyHV-2, Cyprinid herpesvirus 2; *AhR* 1/2, Aryl Hydrocarbon Receptor 1/2; *Cyp1A1*, Cytochrome P450, family 1, member A1; *IFN-γ*, Interferon *γ*; *MX1*, Myxovirus resistance 1; *ISG15*, Interferon stimulated gene 15; *JAK*, Janus Kinase; *TNF-α*, Tumor necrosis factor-α; *MAPK3*, Mitogen-activated protein kinase 3; *IL-4*, Interleukin-4; *Gata3*, Gata binding protein 3; *T-Bet1*, T-box expressed in T cells 1.

**T A B L E S2** Cumulative mortality of gibel carp (*Carassius auratus gibelio*) post-challenged with CyHV-2 via ‘per-gill’ method for 14 days

| **Groups** | **Concentration** | **Days post infection (dpi)** | | | | | | | | | | | | | | **Cumulative mortality (%)** |
| --- | --- | --- | --- | --- | --- | --- | --- | --- | --- | --- | --- | --- | --- | --- | --- | --- |
|  |  | 1 | 2 | 3 | 4 | 5 | 6 | 7 | 8 | 9 | 10 | 11 | 12 | 13 | 14 |  |
| CyHV-2 infection | 9.7×10^7^ copies fish^−1^ | 0 | 0 | 0 | 0 | 1 | 0 | 1 | 0 | 1 | 0 | 1 | 0 | 0 | 0 | 50 |
|  |  | 0 | 0 | 0 | 0 | 0 | 1 | 1 | 0 | 1 | 0 | 1 | 0 | 0 | 0 | 50 |
|  |  | 0 | 0 | 0 | 0 | 0 | 1 | 0 | 1 | 1 | 1 | 0 | 0 | 0 | 0 | 50 |
| Control | PBS (0.01M, pH7.2) | 0 | 0 | 0 | 0 | 0 | 0 | 0 | 0 | 0 | 0 | 0 | 0 | 0 | 0 | 0 |

**T A B L E S3** Operational taxonomic units (OTUs) abundance statistics

| **Groups** | **Raw PE** | **Clean PE** | **Raw Tags** | **Clean Tags** | **Effective Tags** | **Effective Ratio (%)** |
| --- | --- | --- | --- | --- | --- | --- |
| Control-1 | 106570 | 104305 | 94759 | 93257 | 91775 | 86.12 |
| Control-2 | 103662 | 101243 | 92431 | 90140 | 89857 | 86.68 |
| Control-3 | 106517 | 104356 | 95448 | 94265 | 93071 | 87.38 |
| CyHV-2 infection-1 | 102272 | 99860 | 91419 | 90598 | 89001 | 87.02 |
| CyHV-2 infection-2 | 114004 | 111257 | 101836 | 100872 | 98411 | 86.32 |
| CyHV-2 infection-3 | 82201 | 80524 | 74364 | 73684 | 72471 | 88.16 |

**T A B L E S4** Alpha diversity of gut microbiota in gibel carp (*Carassius auratus gibelio*) from both control and CyHV-2-infection groups

| **Groups** | **Chao1** | **Simpson** | **Shannon** | **Coverage (%)** |
| --- | --- | --- | --- | --- |
| Control | 926.60 ± 79.31 | 0.58 ± 0.04 | 2.99 ± 1.00 | 0.997371 ± 0.000239 |
| CyHV-2 infection | 985.54 ± 58.08 | 0.69 ± 0.13 | 2.74 ± 0.11 | 0.996889 ± 0.000391 |

**
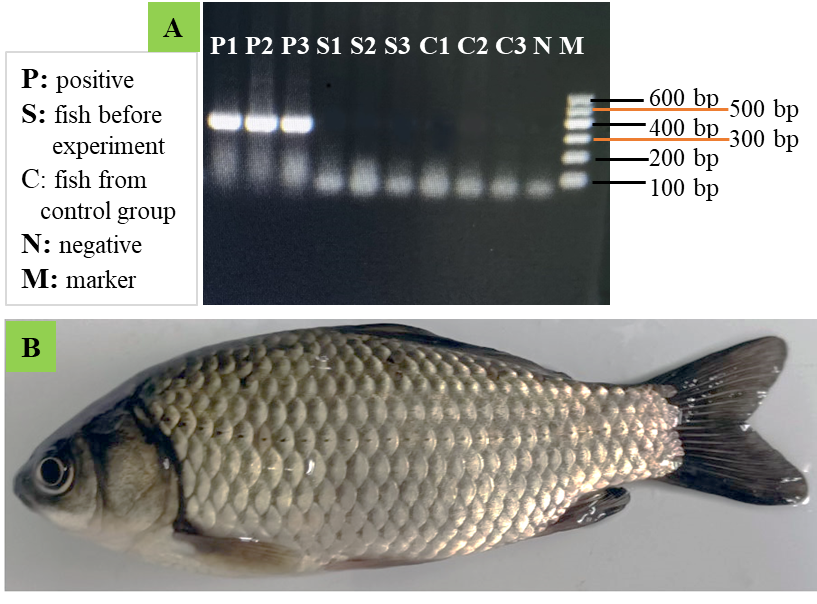
**

**F I G U R E S1** Detection of CyHV-2 in fish before experiment **(A)** and in fish from control group **(B)** using PCR method


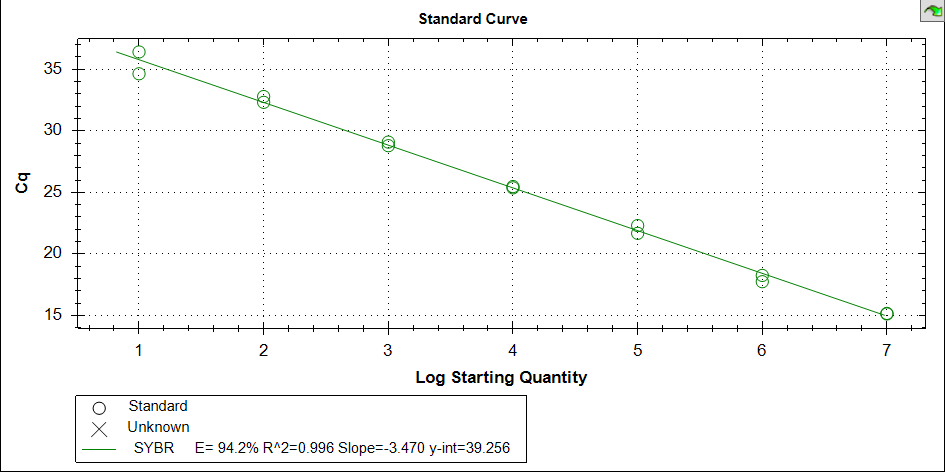


**F I G U R E S2** Standard curve of CyHV-2 load using qPCR method


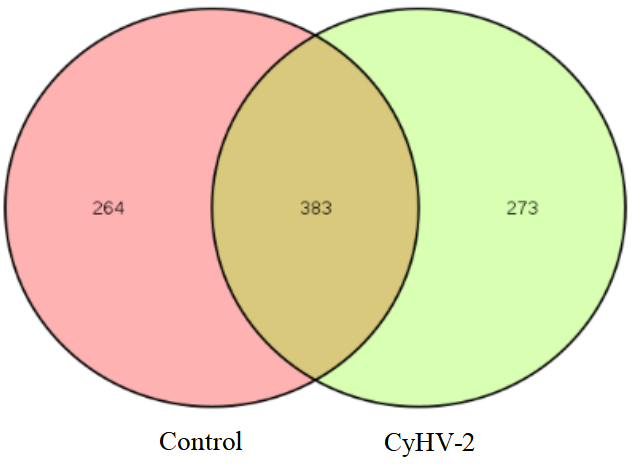


**F I G U R E S3** Venn diagram showing overlapping operational taxonomic units (OTUs) between control and CyHV-2-infection groups

**
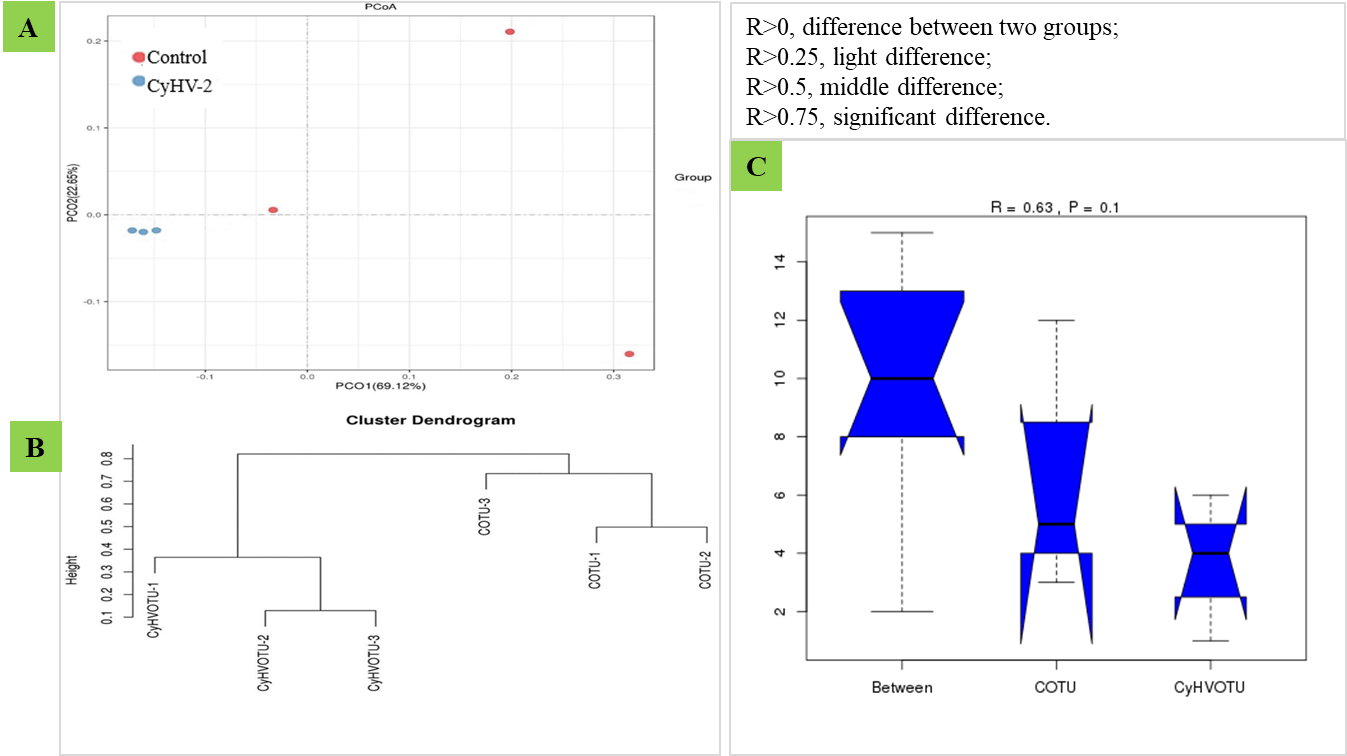
**

**F I G U R E S4** Principle component analysis **(A)**, hierarchical clustering analysis **(B)** by UPGMA and box plot of distance between samples by ANOSIM (Analysis of Similarity) for the gut microbiome patterns between the control (COTU) and CyHV-2-infection (CyHVOTU) groups. The percentage of variation explained by the plotted principal coordinates is indicated on the axes.


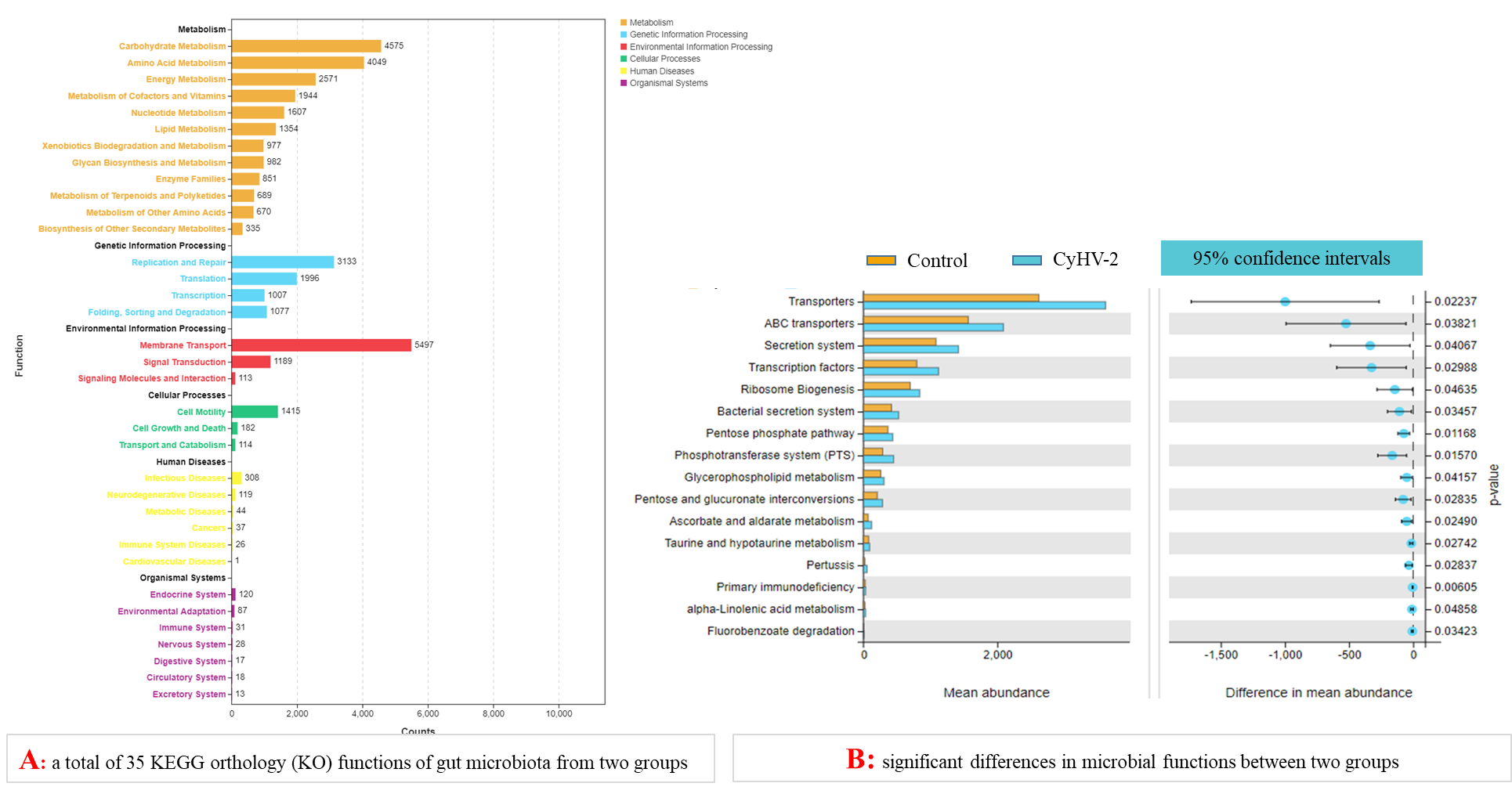


**F I G U R E S5** A total of 35 KEGG orthology (KO) functions **(A)** of gut microbiota predicted using PICRUSt and significantly different function **(B)** from the control and CyHV-2-infection groups
